# Screening for Protein-DNA Interactions by Automatable DNA-Protein Interaction ELISA

**DOI:** 10.1371/journal.pone.0075177

**Published:** 2013-10-11

**Authors:** Luise H. Brand, Carsten Henneges, Axel Schüssler, H. Üner Kolukisaoglu, Grit Koch, Niklas Wallmeroth, Andreas Hecker, Kerstin Thurow, Andreas Zell, Klaus Harter, Dierk Wanke

**Affiliations:** 1 Plant Physiology, Center for Plant Molecular Biology, University of Tuebingen, Tuebingen, Germany; 2 Cognitive Systems, Center for Bioinformatics, University of Tuebingen, Tuebingen, Germany; 3 Center for Life Science Automation, Rostock, Germany; Florida International University, United States of America

## Abstract

DNA-binding proteins (DBPs), such as transcription factors, constitute about 10% of the protein-coding genes in eukaryotic genomes and play pivotal roles in the regulation of chromatin structure and gene expression by binding to short stretches of DNA. Despite their number and importance, only for a minor portion of DBPs the binding sequence had been disclosed. Methods that allow the *de novo* identification of DNA-binding motifs of known DBPs, such as protein binding microarray technology or SELEX, are not yet suited for high-throughput and automation. To close this gap, we report an automatable DNA-protein-interaction (DPI)-ELISA screen of an optimized double-stranded DNA (dsDNA) probe library that allows the high-throughput identification of hexanucleotide DNA-binding motifs. In contrast to other methods, this DPI-ELISA screen can be performed manually or with standard laboratory automation. Furthermore, output evaluation does not require extensive computational analysis to derive a binding consensus. We could show that the DPI-ELISA screen disclosed the full spectrum of binding preferences for a given DBP. As an example, *At*WRKY11 was used to demonstrate that the automated DPI-ELISA screen revealed the entire range of *in vitro* binding preferences. In addition, protein extracts of *At*bZIP63 and the DNA-binding domain of *At*WRKY33 were analyzed, which led to a refinement of their known DNA-binding consensi. Finally, we performed a DPI-ELISA screen to disclose the DNA-binding consensus of a yet uncharacterized putative DBP, *At*TIFY1. A palindromic TGATCA-consensus was uncovered and we could show that the GATC-core is compulsory for *At*TIFY1 binding. This specific interaction between *At*TIFY1 and its DNA-binding motif was confirmed by *in vivo* plant one-hybrid assays in protoplasts. Thus, the value and applicability of the DPI-ELISA screen for *de novo* binding site identification of DBPs, also under automatized conditions, is a promising approach for a deeper understanding of gene regulation in any organism of choice.

## Introduction

DNA-binding proteins (DBPs), such as transcription factors, polymerases, methyl-transferases or histones, play pivotal roles in the regulation of chromatin structure and the control of gene expression. Sequencing of eukaryote genomes disclosed that about 10% of all genes encode potential DBPs. Hence, every higher plant or vertebrate genome harbors over 2000 of these DBP genes [1]–[4]. Despite their importance in many fundamental processes, e.g. during stress or disease, throughout development and in controlling yield or growth, our knowledge on this tremendous number of putative DBPs and their interaction with DNA is limited [1], [2]. In vertebrates, even for the best studied transcription factor classes, i.e., zinc finger domain, basic domain or helix-turn-helix, roughly 20% of all proteins with annotated DNA-binding domain have been characterized experimentally and an accompanying DNA-binding motifs has been reported [2], [5]–[7]. As many classes of DBPs are not (yet) in the focus of investigations, only for approximately 7% of all DBP family members encoded in a eukaryote genome a DNA-binding motif has been described [2].

DNA-binding motifs for monomeric DBPs are usually short (only 4–6 base pairs) and possibly degenerate in their sequence [8], [9] Previous studies revealed that the average size of known DNA-binding domains of DBPs [∼15–30 kDa] is equivalent to six base pairs (bp) [∼20 kDa] contact site of dsDNA [2], [8], [10]–[14]. Minor groove binding proteins, however, were shown to specifically recognize shorter dsDNA motifs of only four bp in length [8]. Consistently, screening of 104 non-redundant DBPs from mouse with protein binding microarrays (PBM) revealed predominantly hexanucleotide (6 mer) binding consensi [10]. Similar results were obtained with PBM technology by screening transcription factors from yeast, where the computationally derived binding consensi were mainly six base pairs in length [15]. However, the same group also reported that several of the proposed binding concensi were longer and represent spaced binding motifs, possibly of transcription factors that can form multimers [15]. This homotypic dimerization of DBPs might probably explain the reports on DNA-binding motifs that are up to 8 turns of the DNA double helix (80 base pairs) in length [5], [16]. For example, the well-characterized prokaryote transcription factor lactose repressor (LacR) can recognize a total of 21 base pairs *in vitro* and *in vivo* [17], [18]. Nevertheless, each monomeric LacR DNA-binding domain is found to bind to 4–5 base pairs of DNA only [18]–[21]. The LacR tetramer, however, forms a deformable entity that is capable of spanning spaced LacR-binding motifs that are up to 401 base pairs apart [17], [22].

The thorough analysis of DBPs and their respective DNA-binding motifs, however, is hindered by a shortage in suitable characterization methods. In addition, it is a prerequisite that these methods need to disclose the full range of DNA-binding preferences for each DBP. Especially, analyses of *in vivo* binding data from yeast and fly suggest that high, medium and low affinity binding sites were of equal importance [23], [24]. The classical approaches for the analysis of protein - DNA - interaction such as Deoxyribonuclease (DNAse) I footprint assay or electrophoretic mobility shift assay (EMSA) all required a given piece of known DNA-sequence to uncover possible protein interaction sites [25], [26]. The subsequent identification of the DBPs that binds to these interaction sites was performed by yeast-one-hybrid screening with a protein expression library [25], [27], [28]. In addition, the specificity of this interaction was again tested in qualitative EMSA using specific DNA-probes and purified proteins [25], [26].

Instead, the increasing knowledge of DBP sequences from genome projects requires the targeted forward molecular analysis that aims at the *de novo* identification of yet unknown DNA-binding motifs [25], [29], [30]. Therefore, acceleration of the entire characterization process is required and, thus, a satisfactory method of choice needs to fulfill most of the criteria for high-throughput methods such as a minimum input of time, cost or labor, a certain robustness of analysis and the possibility of automation [31]. With today's methods of choice like yeast one-hybrid screen, PBM technology or systematic evolution of ligands by exponential enrichment (SELEX) the chance to uncover the DNA-binding motifs of the vast number of putative DBPs seems barely be possible [1], [15], [32].

Although SELEX is a very useful *in vitro* technique, it essentially requires purified proteins, which can be an obstacle that slows down the entire procedure [32]–[36]. Furthermore, SELEX works best with proteins that bind strongly and with a high specificity to their DNA-binding motif [32]–[34], [36]. Chromatin immunoprecipitation (ChIP) provides an invaluable *in vivo* snap-shot of the genome-wide occupancy of possible DNA-binding sites by a particular DBP and has also been used to infer DBP-binding data computationally [9], [25], [29], [30], [37], [38]. However, ChIP is not intended for high-throughput investigation or for the analysis of a yet uncharacterized DBP. ChIP-based techniques are usually performed with proteins that are already known to bind to DNA and possible genomic targets are identified by differential enrichment of specific versus control samples [25], [37]. In contrast, high-throughput yeast one-hybrid screens could support the in-depth characterization of protein-DNA interaction for a given DNA sequence, but are not suited for the *de novo* identification of DNA-binding motifs [25], [27], [28]. In addition, yeast-one-hybrid screens and SELEX are biased towards high affinity binding motifs and do not disclose the full spectrum of binding preferences [25], [28]. At present, only PBM based technologies provide the full range of information about the binding preferences of a protein and, thus, high and low affinity binding sites are identified *in vitro* [1], [11], [39]–[44]. Similar to SELEX, all PBM based methods essentially require highly purified protein for conclusive analyses and subsequent probabilistic inference of a possible DNA-binding motif [1], [11], [39], [41], [42], [44], [45].

Here, we present a new approach with the potential to accelerate the process of *de novo* identification of DNA-binding motifs. It utilizes an optimized double-stranded DNA (dsDNA) probe library for the analysis of DBP - DNA interaction by DNA-protein interaction (DPI)-ELISA [46]. Biotinylated dsDNA probes are individually immobilized on streptavidin-coated wells of a microtiter plate and subsequently probed with HIS-epitope-tagged DBPs. Immunological detection reveals the sequence specific retention of the tested DBP in a well. The DPI-ELISA screen is performed within a few hours in a single microtiter plate, either manually or with standard laboratory automation routines and fulfills the criteria for high-throughput DBP analyses. We applied this approach to the already known DNA-binding proteins *At*WRKY11, *At*WRKY63 and the DNA-binding domain of *At*WRKY33 and revealed their full binding spectra. In addition, we show the applicability of the method for *de novo* DNA-binding site discovery by automated screening of the DNA-probe library with the yet uncharacterized DNA-binding protein *At*TIFY1. The specific interaction between *At*TIFY1 and its GATC-binding consensus was confirmed by detailed *in vitro* studies and validated by in vivo plant one-hybrid analyses.

## Materials and Methods

### dsDPLA (double-stranded DNA Probe Library Algorithm)

We developed the double-stranded DNA probe library algorithm (dsDPLA) for the unambiguous distribution of DNA motifs in the variable region of each oligonucleotide library probe. This algorithm specifically takes the features of double-stranded DNA, such as palindromic sequences or sense and antisense orientation, into account. Within the variable region of the dsDNA probes all possible DNA motifs of a defined length (k-mers) are distributed. In our approach, the k-mers were defined as hexanucleotide DNA motifs (6-mers). We designed the dsDNA probe library in a way that an unequivocal identifiable pattern of positively bound probes is disclosed after specific binding of a DBP to any of the k-mers.

To realize an automatable DPI-ELISA procedure within its technical limitations, dsDPLA needed to meet the following constrains: (a) every possible 6-mer DNA motif is present in the library, (b) the maximal number of dsDNA library oligonucleotides including all control probes is limited to the 384 wells of the microtiter plate, (c) the variable library region of each dsDNA probe is limited to a maximal length of 20 bp, (d) each 6-mer DNA motif is allowed to occur only once per dsDNA probe in either sense or antisense orientation. The dsDPLA is organized as a two-step protocol: It starts with the design of one or more long nucleotide strands (masterstrands) comprising the majority of k-mers via nucleotide-by-nucleotide elongation and the simultaneous insertion of cut-marks to define the preliminary variable region. Subsequently, this partial solution is completed in a second step, where remaining conflicts and designing additional probes *via* a greedy heuristics approach completes missing motifs. For masterstrand construction, dsDPLA implements a backtracking strategy to find possible solutions [47]. The backtracking strategy for the masterstrand ensures that the following constraints are met: (1) a DNA probe sequence contains each k-mer DNA motif at most once, (2) a non-palindromic k-mer DNA motif (sense) and its reverse complement (antisense) do not appear on the same dsDNA probe, (3) if two non-palindromic k-mer DNA motifs appear together on one dsDNA probe (sense), their respective reverse complements (antisense) do not appear together on the same dsDNA probe, (4) two palindromes do not appear on the same dsDNA probe, (5) a DNA probe is at least 15 bp long, (6) a DNA probe does not exceed a maximum length of 20 bp. Constraint (1) ensures that all motifs are covered. Constraint (2) is specific to double-stranded DNA sequences and requires encoding only one orientation of a motif, either its forward sequence (sense) or its reverse complement (antisense) on one probe. Constraint (3) does not allow redundant motif distribution. As palindromic sequences do not differ in their forward and reverse-complement, they need to be placed on separate probes for efficient encoding (constraint 4). Finally, constraint (5) and (6) ensure probe length constraints for a practical library. In case of constraints violation, the search returns and is continued for elongation or a cut-mark is placed. The search stops when a pre-specified length is reached. Then a new masterstrand is started with the goal to integrate yet remaining motifs. For the library used in our manuscript, we chose a solution obtained from three masterstrands each of 1,600 bp lengths. After cutting the obtained masterstrands into a partial library solution, a subsequent construction of completive dsDNA probes is necessary to ensure that the general constraints (a)–(d) are satisfied. The greedy heuristics is used to add missing or ambiguously encoded DNA motifs to new dsDNA probes up to a total of 20 bp. Our heuristic prioritizes dsDNA motifs for the completion of probes according to the number of ambiguously distributed dsDNA motifs. New dsDNA motifs are only added, if they solve the ambiguity. Although the vast majority of dsDNA probes are 20 bp in length, the final library used in this manuscript contains a few probes that possess a shorter variable region, which is due to the second step of library building. With dsDPLA we designed several libraries to increase the probability of finding a reasonable library for the automated DPI-ELISA screen. The bioinformatics of dsDPLA is explained in detail in Protocol S1.

### DNA probe library

**Figure 1:**
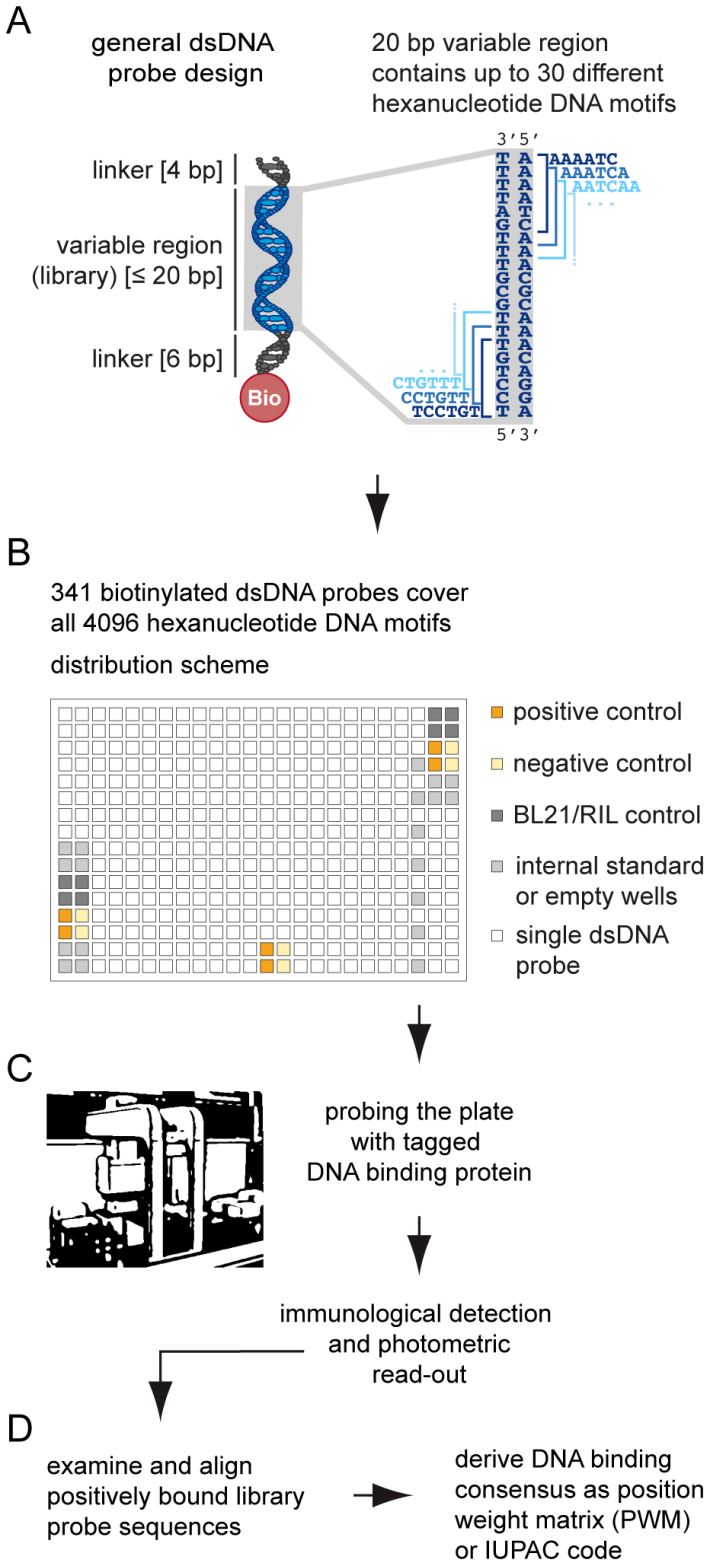
Workflow of the DPI-ELISA screen. (A) Design of the double stranded (ds) DNA probes. Each dsDNA library probe contains a variable library region of up to 20 base pairs, which covers up to 30 different hexanucleotide sequences. Each of the probes is flanked by T/A repeats as linkers. As an example, the double-stranded DNA-sequence of the variable region in dsDNA library probe 18 is shown (right). A total of 30 overlapping, non-redundant hexanucleotides are distributed on both DNA-strands. (B) The individually synthesized DNA probes are annealed and 2 pmol of each dsDNA probe are immobilized in a single well. Hence, each individual dsDNA probe is assigned a specific plate position. Distribution of dsDNA probes on the 384 well plate according to the scheme by using robotics. (C) The DNA coated plate is probed and analyzed according to the DPI-ELISA protocol [46] by using robotics. (D) The photometric readout is normalized to the mean and analyzed to identify positively bound DNA motifs from which a DNA binding consensus is derived.

We designed each dsDNA library probe to contain an invariant linker regions at their plate proximal and distal end, to enhance accessibility and to reduce the risks of sterical hindrance close to the plate surface (Figure 1a). To increase oligonucleotide hybridization efficiency and to avoid the accidental introduction of sequences that are known to form Z-like DNA-conformations *in vitro*, we decided to use adenyl linkers of different length, which results in an asymmetric organization of the dsDNA-probes [48]. The forward strands possess a 6 bp adenyl linker at the 5′ plate proximal end, the variable library region of 11–20 bp and, finally, a distal 4 bp adenyl linker region. Consistently, the reverse complement strands of each DNA probe exhibit a 4 bp thymidine linker at their 5′ distal end, followed by the library region and a 6 bp thymidine linker region proximal to the plate. Only the forward strands of the oligonucleotide probes are 5′ biotinylated, to allow immobilization onto the streptavidin coated microtiter plates. We selected an oligonucleotide array for the 384 well microtiter plate format that consist of 341 dsDNA library probes, 6 positive controls and 26 negative control probes (Table S1 and Figure S1). All single-stranded oligonucleotide probes were ordered from Biomers.net GmbH, Germany. We performed the annealing of the single-stranded oligonucleotides to result in the final dsDNA library probes as was described before [46]. The DNA probes for the detailed analysis of the *At*TIFY1 binding consensus were derived from the positively bound DNA library probe 38 and given in Figure S2.

### Molecular Cloning of *E. coli* expression vectors

The coding sequence of *AtWRKY11 DNA-binding domain* (*DBD*), *AtWRKY33 C-terminal DNA-binding domain* (*cDBD*) [46] and *AtTIFY1* (At4g24470; GenBank accession no. NM_179104) were amplified using cDNA from *Arabidopsis thaliana* flowers as template and gene specific primers from Biomers.net GmbH, Germany (Table S3). The specific PCR products were inserted into the Gateway compatible vector pENTR™/D-TOPO® (Life Technologies, Germany) and transformed into DH5α *E. coli* cells (Stratagene, Germany). After sequencing BP reaction reaction was performed with Gateway® *pET-DEST42* vector according to manufacturer's protocol (Life Technologies, Germany). The *pET-DEST42-AtbZIP63* vector was provided by Kirchler *et al.* [49]. The expression vectors containing a C-terminal His-epitope were transformed into *E. coli* expression strain BL21/RIL (DE3) (Stratagene, Germany). As negative control we used BL21 cells transformed with *pET-DEST42-empty* without *ccDB* cassette [46].

### The automatable DPI-ELISA screen

Proteins were expressed and extracted according to Brand *et al.* [46]. After detection of the HIS-epitope-tagged proteins by western blot analysis protein extracts were used for DNA-protein interaction - enzyme linked immunosorbent assay (DPI-ELISA) screen. Native crude *E. coli* protein extracts of recombinant *Arabidopsis thaliana* WRKY11 DBD, WRKY33 cDBD, TIFY1 and bZIP63 were used for DPI-ELISA as was described [46] (for detailed description of the routine see Figure S1). As negative controls we used protein extract from cells transformed with *pET-DEST42-empty*, wells without DNA probes and W2m DNA (TTacCC) probed with protein extract from cells transformed with *pET-DEST-WRKY11 DBD* [50]. As positive control we used the W2 DNA (TTGACC) probed with protein extract from cells transformed with *pET-DEST-WRKY11 DBD* [46], [50]. The streptavidin-coated 96 and 384 well microtiter plates were obtained from Greiner-Bio-One, Germany. For detailed binding studies of *At*TIFY1 the protocol of Brand *et al.* [46] was applied.

For automated DPI-ELISA screening, we used a BIOMEK FX laboratory automation workstation (Beckman Coulter Inc., Fullerton, California) equipped with single pod and 96-well pipetting head. We almost fully automated both, the distribution of the dsDNA probe library from 96-well stock plates to the 384-well working plates and the entire processing of the DPI-ELISA screening procedure in these 384-well working plates (Figure S1) by using standard BIOMEK control software. Only two steps still require personal surveillance: One, the thorough removal of the washing buffer in the very last wash step, as residual buffer might result in false readout. Two, the decision, when to stop the HRP reaction (Figure S1), is made after manual inspection. The automated DPI-ELISA screening procedure lasted around 5 h, which excludes the time for color development of the HRP reaction product; approximately the same time is required for manual processing.

### Data evaluation of the DPI-ELISA screen

All data analyses were done with simple spreadsheet analyses or by using online tools. The relative normalized absorbance is calculated from the raw absorbance values of each probe on a 384 well microtiter plate relative to the average of the absorbance values of all wells probed with the protein extract of interest. All probes, including positive and negative controls as well as all the library probes, were rank sorted according to their relative normalized absorbance values, which results in S-plot diagrams. The confidence interval and the borders of significance were defined as twice the standard deviation (2σ) of the average relative normalized absorbance values (*p* = 0.05) [51]–[53]. The probes with a relative normalized absorbance above the significance threshold were valued positive (Table S3, Table S4). All positively ranked DNA probe sequences were analyzed with the online tool Discriminative DNA motif Discovery (DREME, version 4.8.1) to identify the DNA-binding consensus [54]. The forward positive sequences were used as query. The forward 341 DNA probe library sequences were used as comparative sequences with settings as default. The WebLogos were created with WebLogo version 3.3 [55].

For the detailed analysis of *At*TIFY1 by standard DPI-ELISA first the normalized absorbance was calculated, which is the measured absorbance relative to the average absorbance of wells probed with the empty vector control (Figure S2). Second, the relative binding in percent was calculated by dividing the normalized absorbance by the average absorbance value of DNA probe 38. Finally, the average and the absolute error of the relative absorbance were calculated.

### Plant one-hybrid

For protoplast one-hybrid assays different sets of effector and reporter plasmids were generated: We adopted the previously published Gateway® compatible *p35S-GAD-GW* destination vector, which was successfully used for plant two-hybrid assays [56], to construct GAL4_AD_-effector plasmids. Subsequently, appropriate *AtTIFY1* ENTRY-clones were used for recombination into *p35S-GAD-GW* destination vector.

The luciferase (LUC)-reporter plasmids were generated on the basis of the well-established *pFRK1-LUC-NOS-At2g19190* control vector (GenBank accession no. EF090416) [57]. To generate *p4x38-LUC-nos* and *p4x38m2-LUC-nos*, the *FRK1* promoter was replaced with a synthetic promoter by restriction/ligation of a *Hin*dIII/*Nco*I fragment. The synthetic promoter fragments, which were ordered at Eurofins MWG Operon, Ebersberg, Germany, contained the minimal Cauliflower Mosaic Virus 35S (CaMV35S) promoter [58], [59] and a 4x repeat copy of the respective library DNA-probe (Text S1). A *p35Smini-LUC-nos* vector was generated by restriction digest of *p4x38-LUC-nos* with *Xho*I/*Sal*I to remove the 4×38 fragment 5′ of the minimal CaMV35S promoter and by subsequent ligation of the compatible ends.

The protoplast transfection of effector and reporter plasmid combinations by PEG-mediated transformation was carried out as described before [60]–[62]. Each replicate experiment reaction was conducted in microtiter plates with ∼200000 protoplasts per well. Luciferase (LUC) activity in the transfected *Arabidopsis thaliana* Col-0 was measured for 90 min. To reduce artifacts due to equilibration after addition of luciferin, average LUC activity was calculated 10 min after start of the reaction until the end of the measurement [80 min total]. Relative light units (RLU) of two independent experiments and a total of 13 replicates were normalized to *p35S-GAD-GW* and *p4x38-LUC-nos* co-transfected control protoplasts (Table S5). The luminescence was determined using the Ascent FL plate reader (Thermo, Germany). Statistical analysis was performed by pairwise t-test.

## Results and Discussion

The purpose of our DPI-ELISA screen is the *de novo* identification of DNA-binding consensi for any given DBP. As DNA-binding motifs for monomeric DBPs seem to be about six base pairs in size [2], [8], [10]–[14], we decided to use a defined dsDNA probe library for the identification of hexanucleotide DNA motifs (6 mer) on a microtiter plate of 384 wells by DPI-ELISA (Figure 1). Therefore, a library of specific dsDNA probes had to be designed, in which all possible 4096 hexanucleotides are distributed in at least two probes to ensure unambiguous DNA-motif identification. We developed a new algorithm that was optimized for dsDNA probe design of a variable library region (dsDPLA; Protocol S1). Our algorithm *ab initio* takes the reverse complement DNA sequences into account, which means a variable region of only 20 bp covers up to 30 different hexanucleotides (Figure 1A). We next added polyadenyl linkers to the 5′ and 3′ end of the variable library region of the dsDNA probes to avoid clashes during the hybridization of single stranded oligonucleotides and to ensure optimal accessibility of the DNA for DBPs close to the plate's surface (Figure 1A) [48]. Although several possible library solutions were revealed by our dsDPLA (Protocol S1), we finally decided to synthesize a dsDNA probe library of 341 specific dsDNA probes (Figure 1B), which left sufficient space for internal controls and standards: To assure the quality of the assay, the final probe array on the microtiter plate included six positive and 26 negative controls (Figure S1). Next the library was probed with a given His-epitope-tagged DBP. The subsequent immunological detection of retained DBP, which was bound to the immobilized dsDNA library probes, by photometric detection (Figure 1C) was performed according to an automated DPI-ELISA screen protocol (Figure S1). Afterwards we ranked all probes, including positive and negative controls as well as all the library probes, according to their relative normalized absorbance values in order to identify positively bound library probes (Figure 1D). As the sequence and plate position of each individual dsDNA library probe is known, we can readily derive the DNA-binding consensus from the sequences of positive dsDNA probes with DREME motif discovery [54] (Figure 1E).

**Figure 2:**
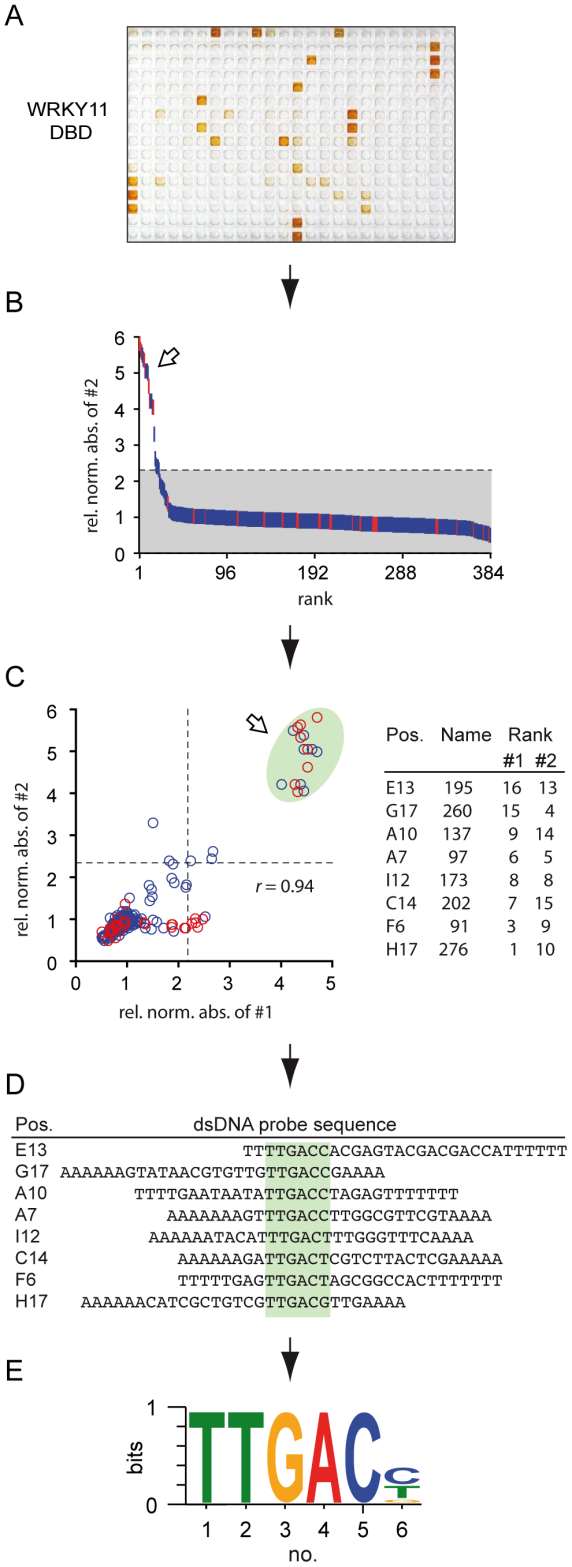
Proof-of-principle experiment using the WRKY11 DNA-binding domain (DBD). (A) Two individual DPI-ELISA screens were performed with WRKY11 DBD; a plate scan of replicate #2 is shown. (B) All 384 probes of the plate replicate #2 were hierarchically ranked according to their relative normalized absorbance values. : positive and negative controls, : dsDNA library probes, ???: indicate positive probes. Background shading indicates confidence interval (p = 0.05) (C) Comparison of the two biological replicate DPI-ELISA screens with WRKY11 DBD. Relative normalized absorbance values of replicate #1 (x-axis) and replicate #2 (y-axis) were plotted against each other to demonstrate the high reproducibility (left). : positive and negative controls, : dsDNA library probe, : indicate positive probes. **- - -**: borders of significance (p≤0.05). Probe name, plate position and rank of positively bound dsDNA library probes that clustered with the positive control probes are given for replicates #1 and #2 (right). (D) The positive dsDNA probe sequences were aligned according to DREME [54]. Pos.: plate position. Finally, the DNA-binding consensus was derived (E).

To validate our automated DPI-ELISA screening strategy with the analysis of a well-characterized DBP, we performed the described routine with the WRKY11 DNA-binding domain (DBD), a GCM1-FLYWCH superfamily member from *Arabidopsis thaliana* [46], [50], [63]–[65] (Figure 2). WRKY11 DBD bound several dsDNA probes with high to low affinity (Figure 2A). It is noteworthy, however, that the vast majority of the dsDNA probes were not bound by WRKY11 DBD, which underlines the overall high sensitivity of the DPI-ELISA and the specificity of the library itself (Figure 2A). The relative normalized photometric absorbance values of all library probes including positive and negative probes were hierarchically sorted to identify significantly bound dsDNA probes compared to the background signals (*p*<0.05) (Figure 2B). All positively bound dsDNA library probes clustered with positive controls (Figure 2B). We observed a high degree of consistency in DPI-ELISA screens for independent biological replicates: two independent DPI-ELISA screen replicates were probed with WRKY11 DBD extracts and accurately revealed similar absorbance values for each of the dsDNA probes with high reproducibility (*r* = 0.94) (Table S3) (Figure 2C). Our results with the DPI-ELISA screen are well comparable to independent probing of protein binding microarrays (PBMs), which analyze several ten thousands of probes and display a similar degree of reproducibility [1], [11], [39], [40]. Out of the 341 library dsDNA probes eight were significantly and reproducibly ranked as positively bound (Figure 2C). A sequence alignment disclosed that seven of these probes perfectly identified the well-known high affinity W-box TTGACY binding consensus for WRKY11 [46], [50], [64]–[67]. However, a TTGACG binding site was also revealed as a high affinity binding motif for WRKY11 DBD (Figure 2D; dsDNA library probe 276 at position H17), which does not fit the stringent TTGACY W-box consensus. Hence, also infrequent binding motifs are discovered by using the DPI-ELISA screen, which is not possible with other methods such as SELEX or PBM, where the DNA-binding consensi are inferred either from sequence reads or from probe sets by probabilistic analyses, respectively [25], [32], [33], [35].

The advantages of standard ELISA-based approaches are their applicability for laboratory automation. Also this new approach of a DPI-ELISA screen benefits from the existing repertoire of tools and machines that are propped to suit the plate based formats of an ELISA. In our case, the successful automation of the DPI-ELISA screen accounts for the overall high reproducibility between two biological replicates. Manual pipetting of the entire DPI-ELISA screen is still possible, but results in a much higher variability of the background due to less accuracy in pipetting 384 well microtiter plates (data not shown). However, positively bound library probes will be recovered at high reproducibility, while background signals will fluctuate in intensity between replicates.

**Figure 3:**
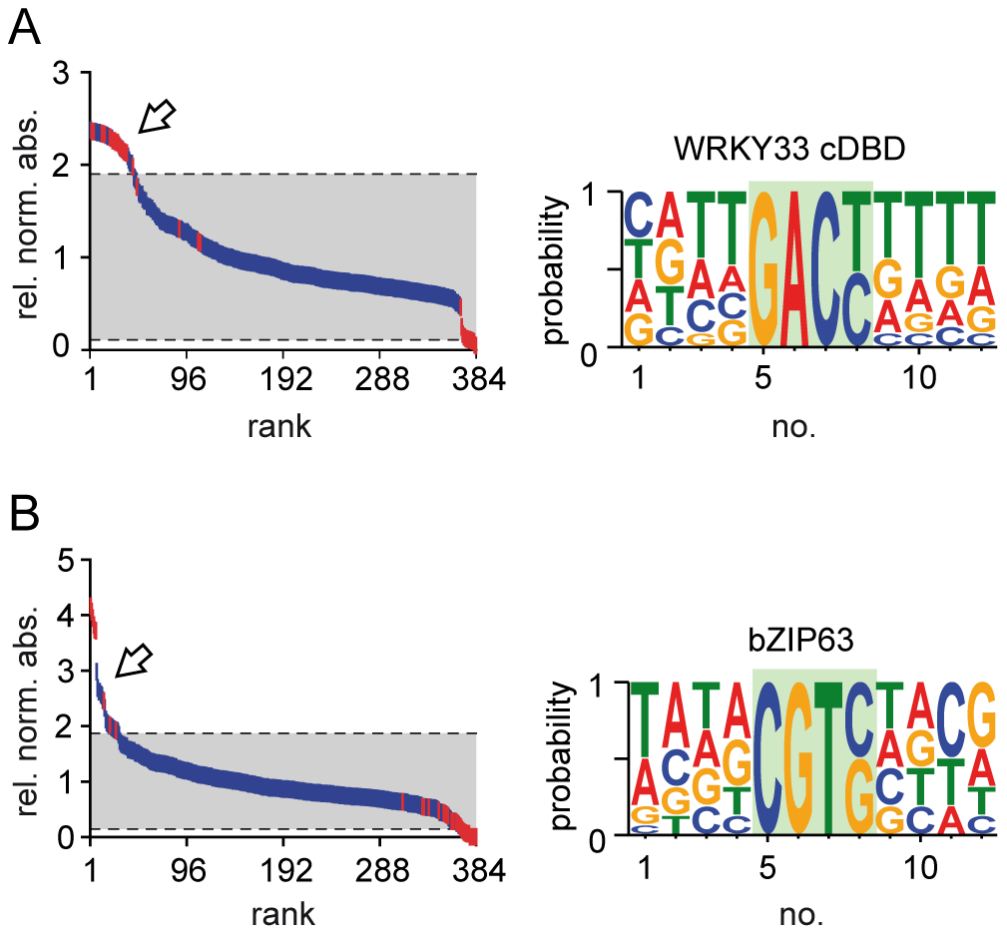
Analysis of the DNA-binding motifs of two different DNA-binding proteins. The results of the DPI-ELISA screen of WRKY33 C-terminal DNA-binding domain (cDBD) (A) and bZIP63 (B) are shown. All 384 probes were hierarchically ranked according to their relative normalized absorbance values. : positive and negative controls, : dsDNA library probes, : indicate positive probes. Background shading indicates confidence interval (p = 0.05). Alignments of positive dsDNA probes were used to deduce the binding consensus next to the graph.

To further validate the DPI-ELISA screen, we conducted this method with DBPs from different protein families. We decided to use representatives of transcription factor families that are distinguished by their different DNA-binding domain architectures: another Zn-finger like WRKY protein, a leucine zipper bZIP protein and a GATA-type Zn-finger protein. First, we analyzed the C-terminal DNA-binding domain of *At*WRKY33 (*At*WRKY33 cDBD) by DPI-ELISA screen. *At*WRKY33 cDBD shares homologies with the distantly related *At*WRKY11 DBD [46], [50], [63], [64], [68], [69]. It is assumed that WRKY family members specifically bind to varying DNA motifs but disclose a common binding consensus core [46], [50], [64], [65], [69]. In our DPI-ELISA screen, *At*WRKY33 binds to a more degenerate W-box consensus with an invariant ‘GACY’ core that, indeed, overlaps with the known W-box consensus [68] (Figure 3A, Table S4). Second, we evaluated the interaction of *At*bZIP63 with its high-affinity C-box ‘GACGTC’ [46], [49], [70]–[72]. Our analysis revealed that *At*bZIP63 recognizes also other motifs besides the C-box and that the binding consensus ‘DCGTS’ is shorter and more degenerate than previously thought (Figure 3B, Table S4).

**Figure 4:**
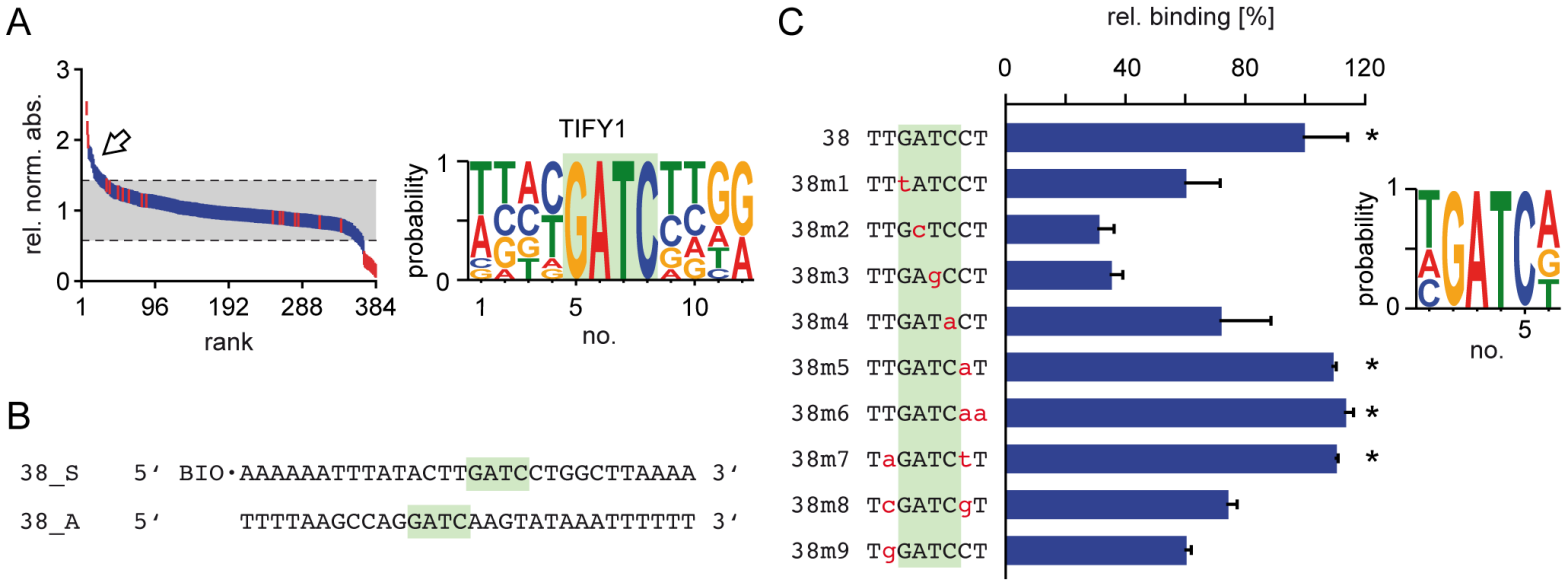
Identification of a DNA-binding motif for *At*TIFY1. (A) All 384 probes of the *At*TIFY1 DPI-ELISA screen were hierarchically ranked according to their relative normalized absorbance values. : positive and negative controls, : dsDNA library probes, : indicate positive probes. Background shading indicates confidence interval (p = 0.05). Alignments of positive dsDNA probes were used to deduce the binding consensus next to the graph. (B) Sequence of the significantly bound dsDNA probe 38 that was chosen for further studies of *At*TIFY1 - DNA-interaction. (C) Analysis of the *At*TIFY1-binding motif by sequential mutagenesis of the dsDNA probe 38 by quantitative DPI-ELISA. Mutagenesis of the identified *At*TIFY1 DNA-binding motif results in a altered relative signal intensity of *At*TIFY1 to the DNA in DPI-ELISA experiments. Relative binding values [%] for the mutated probes are given in comparison to the non- mutagenized dsDNA probe 38. Error bars represent the absolute error of two technical replicates. Significantly bound probes (p<0.05) are indicated by asterisks and are used for the binding consensus that is given next to the graph.

Finally, we used the DPI-ELISA screen to reveal the DNA-binding consensus of a yet uncharacterized putative DBP, *At*TIFY1. As a member of the GATA family it was speculated that *At*TIFY1 might possibly bind to a GATA-like consensus core [73]–[77]. Indeed we revealed a ‘GATC’ binding core for *At*TIFY1 (Figure 4A, Table S4). To validate this tetranucleotide binding consensus and to show the specificity of the interaction, we arbitrarily selected one positively bound dsDNA probe (library probe 38) from our automated *At*TIFY1 screen for further analysis. We tested 13 mutated versions of library probe 38 by manual pipetting, to identify those sites that are essentially required and critical for *At*TIFY1 binding (Figure 4B). Single base pair mutations affecting the palindromic GATC motif and especially its invariant AT core reduced binding drastically (Figure 4C). We confirmed that *At*TIFY1 is capable of binding the GATA-family core GATA *in vitro*, but with significantly less affinity when compared with the original dsDNA library probe 38. *At*TIFY1 bound with highest affinity to the palindromic TGATCA hexanucleotide motif (Figure 4C; Figure S2).

Previous publications demonstrated already that the quantitative and qualitative DPI-ELISA binding data were highly reproducible by additional *in vitro* binding studies [46], [49], [78]–[81]. For example, the specific binding of *At*bZIP63 and *At*WRKY11 to their known DNA-binding motifs were validated in classical EMSA [46], [49]. However, the pivotal question that arises from our studies is whether our results obtained by *in vitro* techniques could also be reproduced *in vivo*.

**Figure 5:**
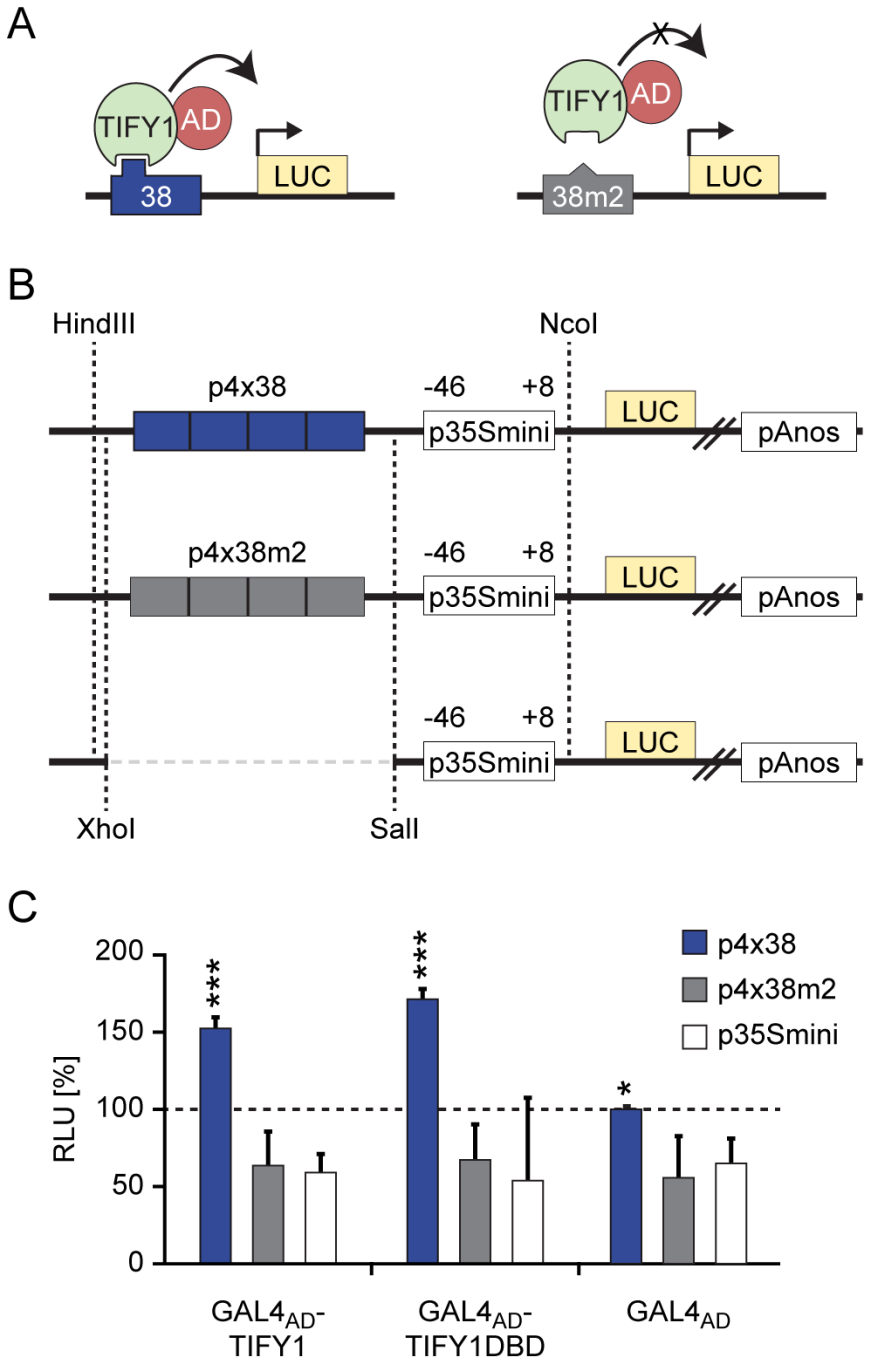
In vivo binding of AtTIFY1 to its GATC-motif. (A) Schematic overview of the plant one-hybrid assay. *At*TIFY1 was translationally fused to the GAL4 activation domain (hybrid effector protein) to test its ability to bind to different DNA motifs within the three different luciferase (LUC) reporters. GAL4_AD_-TIFY1 binding to the 38 probe will induce LUC activity. The same hybrid effector will not bind to the mutated 38m2 probe and background levels of LUC will be recorded. (B) Map of the three different luciferase reporter constructs. Two of these reporters contain the 4x repeat copy of either library DNA-probe 38 or its mutated 38m2 probe (Text S1). All three reporters contained the minimal CaMV35S promoter (p35min) [58], [59], the luciferase coding sequence (LUC) and the NOS terminator (pAnos). (C) Relative luciferase activity of the different reporter constructs in a protoplast one-hybrid assay. *Arabidopsis thaliana* mesophyll protoplast were co-transfected with the different effector and LUC reporter constructs. Relative LUC activity was quantified from two independent experiments [n = 13] and normalized to protoplasts co-transfected with GAL4_AD_ and *p4x38-LUC-nos* [100%]. Error bars indicate the standard deviation of the mean. Asterisks mark combinations of hybrid fusion proteins that mediate significantly increased LUC reporter activity over the background. Protoplasts co-transfected with *p4x38-LUC-nos* and GAL4_AD_-TIFY1 or GAL4_AD_-TIFY1DBD show significant higher LUC activity (*** p≤0,01) than *p4x38-LUC-nos* co-transfected with GAL4_AD_ (*p≤0,05). Furthermore, the mutated luciferase reporters *p4x38m2-LUC-nos* and *p35mini-LUC-nos* co-transfected with any of the three reporter constructs gave relative signals, which are indistinguishable from the background.

Therefore, to validate our findings on *At*TIFY1 - GATC DNA-motif interaction and its specificity *in vivo*, we adopted the principle of the yeast one-hybrid approach in transiently transfected protoplasts of *Arabidopsis thaliana* (Figure 5A). In yeast one-hybrid assays, a candidate transcription factor is translationally fused to the activation domain (AD) of GAL4 to form a hybrid effector protein [25], [27], [28]. Suitable vectors for such an analysis that express GAL4_AD_-hybrid effectors in plant cells were recently published for the plant two-hybrid assay [56]. In our case, upon specific protein-DNA interaction between the hybrid effector and its respective DNA-binding motif, luciferase (LUC) reporter gene activation is mediated by the GAL4_AD_-fusion (Figure 5A). Three different promoter LUC reporter constructs were generated (Figure 5B) and individually co-transfected with one of three GAL4_AD_-effector constructs that express either the full-length *At*TIFY1 protein or the GATA-type DNA-binding domain of *At*TIFY1 (*At*TIFY1-DBD) fused to GAL4_AD_, or only GAL4_AD_ under the control of the constitutive CaMV 35S promoter (Figure 5C).

Consistent with our *in vitro* DPI-ELISA data, *At*TIFY1 hybrid fusion proteins significantly induced LUC activity in a sequence specific manner (Figure 5C). Both, full-length *At*TIFY1 and *At*TIFY1-DBD, GAL4_AD_-hybrid proteins bound to the GATC-motif contained in the library probe 38 specifically. In contrast, the mutated GcTC-motif in the *p4x38m2-LUC-nos* reporter revealed only background LUC activity (Figure 5C). These data indicate that the newly discovered (T)GATC(A) motif is crucial for *At*TIFY1 binding *in vitro* and *in vivo*. Furthermore, the mutation of just one nucleotide in the GATC-core was sufficient to decrease the binding affinity of *At*TIFY1 to DNA drastically, as indicated by our previous DPI-ELISA analyses (Figure 4C).

Interestingly, we also found significant and GATC-motif specific *p4x38-LUC-nos* reporter activation in our GAL4_AD_ controls (Figure 5C), although it was shown before that the GAL4_AD_-effector alone is not capable to bind to DNA and does not contribute to reporter gene activation *in planta* [56]. This can probably be explained by endogenous *At*TIFY1 or other GATA-family members that activate gene expression *via* the functional GATC-motif in the homologous context.

Our *in vitro* DPI-ELISA data and *in vivo* plant one-hybrid analyses revealed a short DNA-binding consensus for the GATA-family transcription factor *At*TIFY1. However, recently published PBM and SELEX data proposed a 30 base pair DNA-binding consensus for mouse PAX4, also a GATA-family transcription factor [5], [16]. In vast contrast, PAX4 was previously shown to bind a pentamer DNA-motif by classical DNA-binding studies [82]. Also other studies on GATA-transcription factors, such as human GATA-1, demonstrated that only short DNA motifs are recognized [83], [84]. In contrast, a much longer 18 base pair DNA-binding consensus was revealed for mouse GATA-1, which is orthologous to GATA-1 in human [5].

These discrepancies are not restricted to GATA-type transcription factors, but seem to be a more general observation: Methods that require extensive bioinformatics to infer a DNA-binding consensus might possibly foster a misleading view on DNA-protein interaction, as the probabilistic methods might prefer longer motifs over the short and abundant ones.

Our current view, which is based on classical EMSA, one-hybrid assays or DPI-ELISA data, supports the idea that neighboring short DNA-binding motifs are functionally organized as *cis*-regulatory modules and are bound by different DBPs possibly in a cooperative manner. Thus, higher order complexes are formed locally, which consequently leads to the formation of long DNA sequence entities. Such *cis*-regulatory modules are conserved through evolutionary time and difficult to separate into individual motifs by bioinformatics [15], [85]–[88], which might be the reason for their co-identification in probabilistic ChIP, SELEX or even PBM analyses.

For example, the High-mobility group B (HMGB) proteins are minor groove binding proteins that bind duplex DNA with low sequence specificity [89], [90]. Yeast Nhp6 is such a HMGB protein that is involved in the positioning of TATA-binding proteins in the proximal promoter and is known to form homotypic multimers that bind up to 11 base pairs [90]–[93]. Each Nhp6 monomer, however, contacts only 5–6 base pairs and sequence recognition is mainly due to DNA-shape readout [8], [93]. In contrast, recently published analyses report an Nhp6 binding consensus that is at least twice as long as the previously reported size (21 base pairs) and contains an invariant TATATA motif core [5], [16]. It is tempting to speculate that this long Nhp6 binding consensus results from probabilistic enrichment of the TATA-motifs in close proximity of the actual Nhp6 binding consensus, which remained cryptic and was not disclosed in the recent analysis. To rule out the possibility of reports on erroneous or artificial DNA-binding motifs, probabilistic methods like SELEX, ChIP or PBM need to be verified independently by a second approach, e.g. by EMSA or DPI-ELISA.

As pointed out before, DNA-binding motifs for monomeric DBPs or known DNA-binding domains appear to be short and possibly degenerate in their sequence [2], [8]–[14]. We are aware that DBPs exist that contain more than one DNA-binding domain and each of the domains might contribute independently to the binding specificity of the full-length proteins. A prominent class of multidomain DBPs that can bind to long DNA-motifs are the TAL-effectors from bacterial plant pathogen species, which DNA-binding domains wind along the double-helix and make contact to dinuclotides each [94]–[98]. Of course, such multidomain DBPs will probably recognize longer DNA-motif - possibly a spaced dyad. However, we have shown that the binding preferences of monomeric DNA-binding domains can readily be assessed by our DPI-ELISA screen.

## Conclusion

Here we could show the quick and economic identification of DNA-binding motifs by DPI-ELISA screen that is potentially applicable to any given DBPs irrespective of the DNA-binding domain architecture. We determined and validated the DNA-binding core of three DBPs and identified the DNA-binding consensus of one so far uncharacterized DBP. These results demonstrated the reliability of the designed dsDNA probe library that was based on hexanucleotide binding motifs. It is possible, however, to generate other libraries with the presented algorithm for the unequivocal distribution of any k-mer, for example to analyze longer dsDNA probes (Protocol S1). The DPI-ELISA protocol is not restricted to bacteria expressed DBPs from *E. coli*, but applicable for the qualitative and quantitative analyses of the DNA-binding properties of any given protein independent from the expression system [46], [78]–[81], [99].

As some DBPs might require post-translational modifications for binding to DNA, eukaryotic expression systems such as HeLa or insect cells might be favorable for expression and protein extraction [81]. To improve the sensitivity of the system, alternatives to colorimetric detection like ECL luminescence or even alternative detection tags, such as DBP fusions with luciferase or with fluorescent proteins, are currently under exploration. To the best of our knowledge, the presented DPI-ELISA screening routine allows for the first time the screening of DNA-protein in an automatable manner with standard laboratory equipment. The DPI-ELISA screen is a significant improvement and logical consequence to the quantitative DPI-ELISA and its prospects, as was shown previously [46], [49], [78]–[81], [100]. Hence, areas of application for the high-throughput DPI-ELISA in genome research and systems biology in its automatable form are apparently broad and harbor a large potential for customization or modification according to personal needs and requirements.

Supporting Reference [101].

## Supporting Information

Protocol S1 **Detailed description of dsDPLA.**
(DOCX)

Figure S1 **DPI-ELISA screen details.** The presented DPI-ELISA screening procedure adapted to the 384 well microtiter plate format took about 5 hours, if the dsDNA probes were at hand. To allow for high-throughput application the system was based on a 96-channel pipette head. The general pipetting scheme is shown (top left). Accordingly, the dsDNA probes were stored in four 96 well plates (I–IV). For a single DPI-ELISA screen we used 1 pmol of each dsDNA probe. After DNA immobilization, washing and blocking the protein binding reaction was performed. According to the pipetting scheme the protein extracts were served in a 96 well plate and distributed on the 384 well DPI-ELISA screen plate. After protein binding, washing and antibody binding four thorough wash steps are required with brief drying of the inverse plate on paper after the last. Finally, the substrate was added and stopped latest after 45 minutes according to the visual impression. The final screen layout is shown (bottom center).
(EPS)

Figure S2 **Detailed analysis of TIFY1 DNA-binding motif.** The DPI-ELISA results of TIFY1 with 13 different versions of the dsDNA probe 38 are shown (a). The DPI-ELSIA results are normalized to the background signals. Absolute errors of two (b) or three (c) technical replicates are shown. Highlighted in red are changed nucleotides; highlighted in grey is the identified binding core consensus.
(EPS)

Table S1 **Library dsDNA probes sequences.**
(DOCX)

Table S2 **Genes of interest.**
(DOCX)

Table S3 **Raw absorbance data of WRKY11 DBD replicates.**
(DOCX)

Table S4 **Positively ranked dsDNA probes of DPI-ELISA screens.**
(DOCX)

Table S5 **Relative luciferase activity values of plant one-hybrid experiments.**
(XLS)

Text S1 **Promoter sequences of plant one-hybrid reporter plasmids.** Promoter sequences of plant one-hybrid reporter plasmids. Promoter sequences are given in 5′→3′ orientation. Library probe 38 and the mutated 38m2 probe are highlighted in red, the CaMV 35Smini sequence is highlighted in green. Sites for *Hind*III, *Xho*I, *Sa*lI and *Nco*I restriction are not highlighted.
(DOCX)
