# Correction: Screening for Protein-DNA Interactions by Automatable DNA-Protein Interaction ELISA

**DOI:** 10.1371/annotation/57c9046c-1549-445c-8733-d0b2cccc5bb9

**Published:** 2014-01-06

**Authors:** Luise H. Brand, Carsten Henneges, Axel Schüssler, H. Üner Kolukisaoglu, Grit Koch, Niklas Wallmeroth, Andreas Hecker, Kerstin Thurow, Andreas Zell, Klaus Harter, Dierk Wanke

The sixth sentence of the last paragraph of the Introduction is incorrect. The correct sentence is: 

"We applied this approach to the already known DNA-binding proteins *At*WRKY11, *At*bZIP63 and the DNA-binding domain of *At*WRKY33 and revealed their full binding spectra."

