# Correction: Screening for Protein-DNA Interactions by Automatable DNA-Protein Interaction ELISA

**DOI:** 10.1371/annotation/25496321-a034-46b0-90e4-5af462e8f068

**Published:** 2014-01-08

**Authors:** Luise H. Brand, Carsten Henneges, Axel Schüssler, H. Üner Kolukisaoglu, Grit Koch, Niklas Wallmeroth, Andreas Hecker, Kerstin Thurow, Andreas Zell, Klaus Harter, Dierk Wanke

There were multiple errors in the legends for Figures 2, 3 and 4 in the online version of the article, and in Figure 2 of the PDF version of the article. The correct legends can be viewed below.

Figure 2: http://www.plosone.org/corrections/pone.0075177.002.cn.tiff

Figure 3: http://www.plosone.org/corrections/pone.0075177.003.cn.tiff

Figure 4: http://www.plosone.org/corrections/pone.0075177.004.cn.tiff

