# Supplementary material for: Screening for Protein-DNA Interactions by Automatable DNA-Protein Interaction ELISA
Source: PLoS One. 2013 Oct 11;8(10):e75177. doi: 10.1371/journal.pone.0075177 (PMC3795721; doi:10.1371/journal.pone.0075177)
Supplement: Protocol S1 — Detailed description of dsDPLA. (DOCX) [file pone.0075177.s001.docx]

# Supporting Protocol S1| dsDNA Probe Library Algorithm (dsDPLA).

DPI-ELISA detects specific protein binding events to a single dsDNA probe. But a single dsDNA probe contains multiple potential DNA binding motifs that might account for the specific interaction found by the assay read-out. For instance, a dsDNA probe of 15 base pairs contains up to 10 different hexameric DNA binding motifs. More generally, a dsDNA probe of length n contains up to (n-k+1) k-mer binding motifs. The algorithm described below constructs a minimized set of dsDNA probes, a *library* that allows unambiguous identification of DNA binding motifs from DPI-ELISA read-out. We start with some definitions used throughout the algorithm descriptions:

**Definitions.** Let the *encoding function* $E$ map a given k-mer p to the subset of dsDNA probes $E\left(p\right)$ that contain p. It is defined as

$$\begin{matrix} E:\Sigma^{k}\to P\left(S_{k,n}\right) \\ E\left(p\right)\mapsto\left\{o\left|p\in D_{k}\left(o\right)\right.,o\in S_{k,n}\right\} \end{matrix}$$

Where a k-mer is based on the DNA alphabet $\Sigma=\left\{A,G,T,C\right\}$ and has a fixed length of k base pairs. Furthermore, let$S(k,n)$ denote a library of dsDNA probes for k-mers with a maximum probe length of n base pairs. Let$P\left(S(k,n)\right)$ be the power set of $S(k,n)$. Finally, let$D_{k}\left(o\right)$ denote the k-decomposition of a dsDNA probe o that is the set of k-mers that appear on o. A dsDNA probe library *encodes* a k-mer p, if the encoding function $E$ maps p to a non-empty subset. It encodes p *uniquely*, if $E$ maps p to a non-empty subset to which no other k-mer is mapped. We say a dsDNA probe library is *complete*, if all k-mers are encoded. We further classify encoded k-mers p by the cardinality of $E\left(p\right)$. If p is encoded only once we use the shorthand *single*, if it is encoded twice it is a *double*.

A k-mer p *coincides* with a k-mer q, if both p and q are encoded on the same dsDNA probe. The set of k-mers with which a given k-mer p shares at least one dsDNA probe is called the *coincidence set* C(p), defined as

$C\left(p\right)=\left\{q\left|p,q\in D_{k}\left(o\right),p\neq q,o\in S_{k,n}\right.\right\}$.

**Algorithm Overview.** Our actual algorithm works in three stages to design a complete and unique library. First, we employ a backtracking algorithm to calculate a *masterstrand*. Simultaneously, the algorithm places *cut-marks* where the masterstrand is cut into dsDNA probes by a second step, which creates an initial but incomplete and ambiguous library. Third, a greedy heuristic amends the incomplete library with additional probes to create a complete and unique library.

**Masterstrands.** We define a *masterstrand* as a theoretical single-stranded nucleotide string that contains every single-stranded k-mer exactly once. This makes masterstrands a very parsimonious starting point to find a complete and unique library. For a graph-theoretical proof for the existence of general k-masterstrands see appendix of this note. On a single-stranded masterstrand, a k-mer and its reverse complement are treated as two different entities by our algorithm, with the exception of palindromic k-mers that are identical to their reverse complement. However, the probes used for the DPI-ELISA are double-stranded and therefore a k-mer and its reverse complement are synonymous in the dsDNA probe library. Consequently, whether a single-stranded k-mer or its reverse complement is added to the masterstrand, the result will be functionally equivalent with respect to the DPI-ELISA read-out. Our algorithm, however, encodes every single-stranded k-mer, with the exception of palindromes, on a *complete masterstrand* twice. Any double-stranded DNA binding motif thereby is identified by a sparse binary pattern, consisting of those two probes that contain the k-mer and its reverse complement.

**Backtracking algorithm.** We decided to use a backtracking algorithm as a flexible method to search masterstrand solutions (11). The algorithm develops a solution incrementally from smaller partial solutions. However, whenever a partial solution violates one of the predefined constraints all its derived solutions will be invalid. Therefore, this partial solution is discarded and the search returns to the valid partial solution that was found before. For the problem of constructing a masterstrand, the backtracking algorithm starts with a single k-mer, and then extends this partial solution one nucleotide at a time. Each extension of the partial masterstrand is equivalent to the addition of a k-mer to the solution. The added k-mer is defined by the last k-1 nucleotides of the strand and the added k-th. Each extension yields a new, larger partial solution, which is then checked for constraint violations.

**Listing 1|** Pseudo code backtracking recursion

void buildMasterstrand(String strand) {

if (isCompleteMasterstrand(strand)) {

print(strand);

return;

}

for each nucleotide base b { //A,T,G,C

newStrand = strand + b;

if (checkConstraints(newStrand)

buildMasterstrand(strand);

}

}

The backtracking approach allows us to define feasible solutions by a set of constraints that every partial solution needs to fulfill. Since our algorithm builds masterstrands incrementally, any valid partial masterstrand, that is any strand that can be extended to a valid masterstrand, must not contain a single-stranded k-mer more than once. However, it may contain its reverse complement instead. Thus, constraint (1) is the only necessary constraint for building valid masterstrands.

**Constraint 1.** A partial masterstrand may contain each single-stranded k-mer at most once.

Under this constraint, the backtracking algorithm above quickly calculates a complete masterstrand. However, not every masterstrand, when cut into dsDNA probes, leads to a valid dsDNA probe library.We require a dsDNA probe library to be *complete* and *unique*. A complete library contains every possible k-mer. It follows immediately that a library consisting of probes from a complete masterstrand has to be complete itself. To achieve a unique solution, that is a solution encoding every k-mer unambiguously, a masterstrand has to fulfill three additional constraints. All of these new constraints govern how hexanucleotides are distributed when the masterstrand is cut into probes by the subsequent step. Per construction, a masterstrand encodes every k-mer twice with the exception of palindromes. These two occurrences of the same k-mer have to be on different dsDNA probes. Thereby, a positively bound DNA-binding protein (DBP) is expected to result in positive read-out for two different dsDNA probes.

**Figure 1**| Situation (a) shows that k-mer p and its reverse complement p are encoded on the same probe. This is equivalent to k-mer p being encoded as a single, and is only problematic if another k-mer on the same strand is also encoded only once. Situation (b) illustrates when the same two probes ambiguously encode k-mer p and q. This is the case when k-mers and their reverse complements for p and q are co-located on the same probes. Situation (c) deals with palindromes encoded by the same probes. As palindromes only offer a single k-mer for encoding, this situation cannot be solved efficiently without violating pasimony. Constraints (2) and (3) prevent situations (a) and (b), while situation (c) is covered by constraint (4).

**Constraint 2.** A non-palindromic k-mer and its reverse complement may not be encoded on the same probe (see Figure 1, case a).

Distributing the same k-mer over different probes is not completely sufficient for an unambiguous masterstrand solution. We also have to ensure that none of the other possible k-mers from the first probe is involved in a binding pattern at the second probe.

**Constraint 3.** If two non-palindromic k-mers appear together on one probe, their respective reverse complements may not appear together on the same probe (see Figure 1, case b).

Constraint (3) together with constraint (2) guarantees that every non-palindromic k-mer appears on a unique subset of two dsDNA probes. This constraint sharply increases the combinatorial complexity, because it creates a large number of dependencies among dsDNA probes. The introduced constraints (2) and (3) deal with non-palindromic k-mers. Palindromes, being identical with their own reverse complement, appear only once within a masterstrand and, thus, in only one dsDNA probe. In contrast to non-palindromic binding motifs, any palindromic binding motif must be uniquely identified by its unary read-out, i.e. only a single probe shows a positive signal for them. It follows, that to ensure unique encoding of all palindromic k-mers, no two palindromic motifs can be placed on the same probe. Moreover, all of the other k-mers on the same probe with a palindome have to be non-palindromic.

**Constraint 4.** Two palindromes may not be encoded by the same probe (see Figure 1, case c).

To check for constraints (2), (3) and (4), our algorithm needs to keep track of individual dsDNA probes while building the masterstrand. A fixed probe length would make it easy to demarcate individual probes. However, dsDNA probes are allowed to vary in length within a specified range. To take advantage of this flexibility, our algorithm inserts *cut marks* into the masterstrand to define the start of new putative probes. Cut marks do not only allow to check for violations of constraints (2), (3) and (4), they also enable the algorithm to actively resolve such violations.

**Listing 2:** Pseudo-code for constraint checks

boolean checkConstraints(String strand, String extension) {

String newKmer = strand.suffix(k-1) + extension;

// constraint 1

for all probes o {

if o.contains(newKmer)

return false;

}

// constraint 2

if (latestProbe.contains(revComp(newKmer)))

return false;

// constraint 3

for all probes o {

if (o.contains(revComp(newKmer))) {

for all kmers h in o {

if (latestProbe.contains(revComp(h))

return false;

}

}

}

// constraint 4

if (isPalindrome(newKmer)) {

for all kmers h in latestProbe {

if (isPalindrome(h))

return false;

}

}

}

Whenever there is a violation of constraints (2), (3) or (4), inserting a cut mark resolves the conflict, because the newly added k-mer then becomes the first on a new dsDNA probe. Resolving conflicts in this way increases the number of dsDNA probes in the library, which quickly becomes counterproductive. Therefore, we introduced a minimum length requirement for dsDNA probes.

**Constraint 5.** A dsDNA probe must reach a specified minimum length.

If the current probe has already reached the minimum length and if there is no valid extension for the incomplete masterstrand, our algorithm inserts a cut mark and starts a new dsDNA probe. The algorithm also inserts a cut-mark when the specified maximum length for dsDNA probes has been reached.

**Constraint 6.** A dsDNA probe must not exceed a specified maximum length.

**Listing 3:** Pseudo-code of cut mark algorithm

void buildMasterstrand(String strand) {

if (isCompleteMasterstrand(strand)) {

print(strand);

exit();

}

if (latestProbe longer than maximum probe length)

strand = insertCutmark(strand);

for each nucleotide base b { //A,T,G,C

newStrand = strand + b;

if (checkConstraints(newStrand)

buildMasterstrand(strand);

}

if (latestProbe has reached minimum probe length) {

strand = insertCutmark(strand);

for each nucleotide base b { //A,T,G,C

newStrand = strand + b;

if (checkConstraints(newStrand)

buildMasterstrand(strand);

}

}

With the described constraints in place, we found that the backtracking algorithm no longer terminates within a reasonable computing time (for a 6-mer library several weeks on a 24 nodes AMD Opteron 2.4 GHz dual-core processor computing cluster, each with 6 GB RAM). This suggests that either there are no complete masterstrands for 6-mers, which fulfill all of the constraints, or there are so few of them that they are unlikely to be found in a reasonable time by the constrained backtracking search. Given that the search space is highly symmetrical, we would assume that there exist no masterstrands for 6-mers that satisfy constraints (2)-(4) simultaneously. On the other hand, we found that the search recurses fairly deep despite of the constraints. The length of incomplete masterstrands that our algorithm calculated as partial solutions supported this. For instance, with the length of a masterstrand for 6-mers at 4101 bases, partial solution sizes ranged within 3703 ± 39 bases for a maximum probe length of 15 base pairs and 3376 ± 39 bases for a maximum probe length of 20 base pairs.

These partial solutions fulfill all of the constraints and are thus very parsimonious: no unambiguously encoded k-mer appears more than twice and no ambiguously encoded k-mer more than once. In addition, many dsDNA probes achieve the maximum length. Therefore, we decided to wrap the backtracking search developed so far into a *multi-start* heuristic: our algorithm calculates a specified number of shorter strands instead of searching the longest possible single-stranded partial solution. So, all partial masterstrands are limited to a specified length and the backtracking search is terminated whenever either the specified length is reached or a specified amount of computing time has expired. Each backtracking search prioritizes the addition of k-mer candidates according to the overall set of k-mers that have not been encoded in the previously computed partial masterstrands. To this end, every k-mer is initially assigned the lowest priority 1. After a partial masterstrand has been calculated, the priority of k-mers encoded in the strand is reset to 1 and the priorities of all other k-mers are increased by 1. The subsequent backtracking step then favors the nucleotide base that corresponds to the candidate with the highest priority. If no highly prioritized k-mer candidates are available, the strand is extended with k-mers of a lower priority. This can lead to k-mers already contained in previous partial masterstrands being encoded again. Such a redundancy increases in library size, but does not violate the given constraints.

**Cutting algorithm.** Once our algorithm has calculated the specified amount of partial masterstrands in the first step, those strands are then cut into putative dsDNA probes. Splitting them at the previously placed cut marks does this. To this end, the cutting step has to repeat an overlap of the last k-1 nucleotides from the previous probe on the masterstrand (see Figure 2). This overlap between probes is necessary to retain all encoded k-mers, because the first k-mer on a probe consists of the nucleotide that was used to extend the partial masterstrand and the last k-1 nucleotides on the previous probe.

**Figure 2**| When cutting the masterstrand into dsDNA probes, the ending nucleotides need to be replicated to not destroy the last appended k-mer.

**Library Completion.** In a final step, we implemented a greedy heuristic that is designed to complete the partial solutions obtained from the multi-start heuristic above. Listing 4 shows the pseudocode for the library completion algorithm, and the text will refer to certain steps mentioned there. In the context of the library completion algorithm, we mean that a k-mer can be preposed or postpositioned when using the term *added to a dsDNA probe*. This is in contrast to the backtracking algorithm, which was restricted to extend probes by adding k-mers to the end of a masterstrand. Now, in the context of a post-processing algorithm, considering k-mers as well as their reverse complements for both prefix and suffix of a dsDNA probe during addition greatly increases the available options for probe extension.

Given a partial solution for a library, two subtasks remain to be solved. The first is to ensure that the library is complete and thus encodes all possible k-mers as binding motifs. The second is to resolve the remaining ambiguities. In step 1 in Listing 1 below, our algorithm creates one dsDNA probe for any k-mer not yet included in the library. The library is then complete and contains the added k-mer as single. This step does not significantly increase the library size as most k-mers so far have been encoded at least once. The algorithm then extends probes less than the maximum length to disambiguate other k-mers. Here, it is important to understand that all k-mers that were encoded uniquely by the masterstrand solution will remain uniquely encoded even if they were added somewhere to newly created dsDNA probes. To illustrate this, consider any two uniquely encoded k-mers. Adding either or both sequences to any other probe does not destroy the discriminability. Because to distinguish both by a DPI-ELISA screen read-out, we only need to analyze the part of the library that encodes for the relative unique pattern, i.e. the part of the library that helps to distinguish between both. By the same argument, the addition of spacer regions to improve protein binding performance is therefore uncritical, as well. This observation therefore allows the algorithm to only focus on those k-mers that are ambiguously encoded.

To optimize disambiguation of already encoded k-mers, the algorithm computes a priority for each ambiguous k-mer h (step 2). The number of ambiguous coincidences determines the priority. k-mers with more ambiguous coincidences are more difficult to encode than others. A high number of ambiguous coincidences imply also a large number of dependencies that tightly constrain the disambiguation of the k-mer, when adding it to a probe. Therefore, it is favorable to add k-mers with more ambiguous coincidences early and, thereby, resolve complex conflicts first. At this point, we cannot expect a perfect overlap of k-1 base pairs when adding a k-mer. Optimal overlap was a key ingredient for the backtracking search, which led to an optimal partial solution. The completion algorithm however progressively relaxes an *overlap size* *requirement* r (step 3), while adding k-mers. Initialized with a required overlap r of k-1 base pairs, r is decreased whenever iteration fails to disambiguate a k-mer (step 5). If the overlap requirement r becomes 0 and no further additions are possible, a new probe is created and the overlap requirement r is reset to k-1 base pairs (step 6). In this way, our heuristic greedily minimizes the number of probes in the library.

To resolve ambiguity between a pair of k-mers within each iteration (step 4), one of them must be added to another probe. Here, all probes can be used that were created by the completion method as well as by the backtracking algorithm. The latter ones were the result of inserting an early cut mark. Each extended dsDNA probe must be short enough such that its extension does not violate the maximum length constraint. In this way, our library completion algorithm creates additional probes only when it is necessary. However, adding k-mers to already existing probes offers another problem. Given ambiguous k-mers p and q, adding p to a different probes o has to ensure that q will not be added to o in the following steps. Otherwise the ambiguity would not be resolved. To this end, we define the *set of ambiguous coincidences* $A(p)$ of p as the intersection of coincidences $C(p)$ with the set $H$ of ambiguous k-mers

$A\left(p\right)=C\left(p\right)\backslash\text{H}$.

Now, for each newly added probe o our completion algorithm maintains an *exclusion list* $X(o)$. If a k-mer is element of $X(o)$, it cannot be added to o. Whenever our algorithm adds k-mer p to o, $X(o)$ is joined with $A(p)$, which then prevents the later addition of any ambiguous coincidence of p. Thereby, the sets $X(.)$ constrain the addition of k-mers to probes. Implementing those constraints by lists enables us to first save memory space because these lists will be sparse and, second, to optimize search by ranking probes with respect to the lengths of $X(o)$ and, thereby, quickly finding those o where appending a k-mer is likely to be successful.

**Listing 4:** Pseudo code for Greed Completion Algorithm

void greedyCompletion(missingKmers, ambiguousKmers) {

/* step 1: code all missing k-mers as singles, each on a new probe */

for each missingKmer h {

probes.add(h);

}

/* step 2: sort ambiguous k-mers in descending number of ambiguous coincidences */

for each ambiguousKmer h {

priority(h) = size(A(h))

}

sort ambiguousKmers by priority in descending order;

/* step 3: add kmers with the largest number of ambiguous coincidences first */

r = 5;

while(r >= 0 and size(ambiguousKmers) > 0) {

/* step 4: for each remaining ambiguous k-mer find a suitable probe with overlap k and concatenate */

for each ambiguousKmer h {

for each probe o {

if (h fits to o &&

o does not exceed maximum probe length) {

if (no ambiguous kmer h2 in o exists, for which h is element of CoincidenceSet(h2))

{

o = concatenateWithOverlap(o, h);

break;

}

}

}

/* step 6: create a new probe if there is no place for the current k-mer */

if (h does not fit to any probe and

r is 0) {

probes.add(h);

}

}

/* step 5: decrease required overlap and continue */

--r;

}

}

**Final library selection.** To compute appropriate dsDNA probe libraries for 6-mers, we ran first the backtracking algorithm starting with each possible hexanucleotide as a seed. For each obtained set of masterstrands, we then applied the cutting algorithm and the greedy completion heuristic to obtain a unique and complete library, encoding each possible 6-mer motif uniquely.

The parameters for the ultimately used solution defined the computation of 3 partial masterstrands, each having a length of 1600 base pairs. The described post-processing algorithm then completed this solution to a complete and unique dsDNA probe library composed of 341 probes, each being less than 20 base pairs long.

***Appendix***

Text provided in this appendix is meant supportive for the understanding of the described algorithms, but not critical for their implementation. It is intended for a mathematical audience with background in algorithms and graph theory. First, we show that masterstrands covering any k-mer exist and therefore a backtracking search, disregarding all constraints, can find a solution. Second, we give a theoretical lower bound for the minimal number of probes as a function of k.

**Proof for Existence of General k-masterstrands.** k-masterstrands, that is a masterstrand computed for k-nucleotides, have two defining aspects: *completeness* (they contain every k-nucleotide) and *maximum parsimony* (they contain each k-nucleotide exactly once). For example, simple concatenation of all k-nucleotides into a single strand fulfills the criterion of completeness. However each concatenation would implicitly include k−1 additional k-nucleotides, which would violate the criterion of parsimony. Our recursive depth first search algorithm extends the strand by one nucleotide at a time. The k-nucleotide added is determined by the strand suffix of length $k-1$ plus the nucleotide by which it is extended. This can be illustrated by a state graph (V, E), its vertices $V=\Sigma^{k-1}$ representing the strand suffix and labelled edges $E=\left\{\left(v_{1},v_{2},l\right)\left|v_{2}=\text{suf}_{\text{k-2}}\left(v_{1}\right);v_{1},v_{2}\in V,l\in\Sigma\right.\right\}$ representing the extending nucleotide (see Figure 3). Each vertex of such a graph always has four direct predecessors and four direct successors. The successors are determined by the extending nucleotide, of which there can only be four. The predecessors depend on the specific sequence of the suffix and also can only differ by a single nucleotide base, the first one. A path in this graph represents the generation of a strand extended by a single nucleotide base per step.

1. Completeness. There is a vertex for each $(k-1)$-nucleotide and each vertex can be reached in $(k-1)$ steps by following the specific sequence of this $(k-1)$-nucleotide. Each vertex has four successors, which determine the last nucleotide of four possible k-nucleotides per vertex. Therefore every k-nucleotide can be contained within a strand generated by a path in such a graph, simply by the path following the edge labels corresponding to the sequence of this k-nucleotide.

2. Parsimony. Each edge represents the addition of a specific k-nucleotide. Thus, to ensure maximum parsimony, the path of a masterstrand may visit each edge only once. The existence of a k-masterstrand is equivalent to the existence of an Eulerian path. Since our graph is connected and each vertex has an equal in-degree and out-degree, an Eulerian path exists, as per proof in (37).


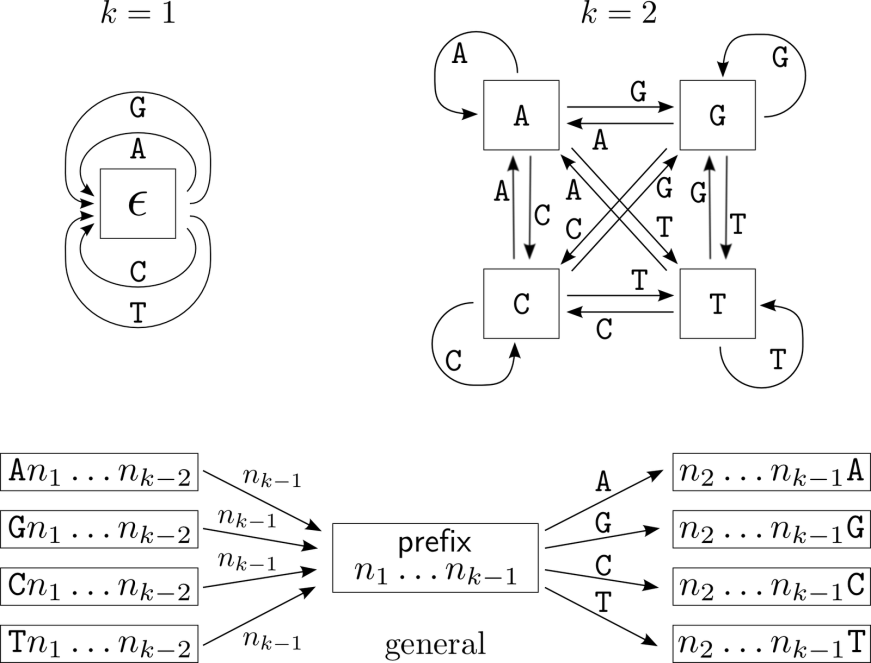


**Figure 3**| Search space represented as annotated graph for k-masterstrands. Node labels represent (k-1)-mers. Each edge represents a state transition equivalent to the addition of a k-mer to the growing masterstrand.

**Lower Bound For Library Sizes.** To evaluate the size of our library solution, we determine a theoretical lower bound for feasible library sizes. According to our objectives, all dsDNA probes should range between a lower and upper number of base pairs in length. Each probe consists of two flanking sequences, described in the algorithm above. The variable sequence of the probes was set to reach a minimum and not exceed a maximum length of base pairs.

Let us consider the case of a hexamer library as an example first, as this was relevant for our project. Each hexamer must be encoded at least once to encode also its corresponding hexanucleotide. Hexanucleotides encoded thereby as singles can only be unique if all other hexanucleotides on the same probe are encoded at least as doubles. This means that for those hexanucleotides both corresponding hexamers must be encoded. Consequently, an optimal solution uses only singles and doubles for encoding the 2080 distinct double-stranded hexanucleotides (ss = single-stranded; ds = double-stranded):

$\underbrace{\frac{\overbrace{4^{6}}^{\text{All possible ss hexamers}}-\overbrace{4^{3}}^{\text{All possible ss palindroms}}}{2}}_{\text{All possible ds non-palindromic hexamers}}+\underbrace{4^{3}}_{\text{All possible ds palindromes}}=2080$.

In general, a probe of length n can contain exactly $n-k+1$ k-nucleotides. Here, these are n-5 hexanucleotides. Probes of length 15, our intended minimal length, therefore contain exactly 10 hexanucleotides. To encode all double-stranded hexanucleotides once, at least 208 probes are needed. If we assume a maximum number of 208 singles, we would have to encode $\frac{9}{10}$ of the remaining hexanucleotides as doubles. If we - optimistically - assume that hexanucleotides can be arbitrarily combined on probes, a unique encoding of all hexanucleotides would require a total of $208+\left\lceil\frac{9}{10}\cdot\frac{2080}{10}\right\rceil=396$ probes of length 15. Of course, this assumption only holds, if the hexanucleotides of every probe overlap perfectly, which is unlikely to be the case and renders this value a lower bound.

Generalization of the example case before to k-nucleotides and probes of length n, the formula for the lower bound for the size of a library can be shown to be

$\left|S_{k,n}\right|\geq\left\lceil\frac{2n-2k+1}{n-k+1}\cdot\left\lceil\frac{d_{k}}{n-k+1}\right\rceil\right\rceil$.

An overview of the minimal possible number of dsDNA probe solutions of our dsDPLA approach with respect to the k-mers and length of the variable library region are given in Table 1.

**Table 1**| Overview of possible library results. Minimal number of oligonucleotide probes needed to distribute a given k-mer on a variable library probe with length n.

| *k / n* | *20* | *30* | *40* |
| --- | --- | --- | --- |
| 4 | 16 | 10 | 7 |
| 5 | 64 | 40 | 29 |
| 6 | 274 | 164 | 118 |
| 7 | 1171 | 683 | 482 |
| 8 | 5042 | 2850 | 1986 |
