# Supplementary material for: Screening for Protein-DNA Interactions by Automatable DNA-Protein Interaction ELISA
Source: PLoS One. 2013 Oct 11;8(10):e75177. doi: 10.1371/journal.pone.0075177 (PMC3795721; doi:10.1371/journal.pone.0075177)
Supplement: Table S1 — Library dsDNA probes sequences. (DOCX) [file pone.0075177.s004.docx]

**Supporting Table S1|** Library dsDNA probes sequences.

| **Name** | **Forward sequence 5’** 🡪 **3’** | **Name** | **Compl. reverse sequence 5’** 🡪 **3’** |
| --- | --- | --- | --- |
| 1_S | AAAAAAGTCTAGTCGTAAAAA | 1_A | TTTTTACGACTAGACTTTTTT |
| 2_S | AAAAAACGGTAAGTCGACAAAA | 2_A | TTTTGTCGACTTACCGTTTTTT |
| 3_S | AAAAAATTACGTTTGTATTTGTAAAA | 3_A | TTTTACAAATACAAACGTAATTTTTT |
| 4_S | AAAAAAACGATACGGTATCTAGAAAA | 4_A | TTTTCTAGATACCGTATCGTTTTTTT |
| 5_S | AAAAAAATCCCACTTCATTCTAAAAA | 5_A | TTTTTAGAATGAAGTGGGATTTTTTT |
| 6_S | AAAAAAATTACGCGGGATCGCCAAAA | 6_A | TTTTGGCGATCCCGCGTAATTTTTTT |
| 7_S | AAAAAAGCTTCGCGCCAGCGGGAAAA | 7_A | TTTTCCCGCTGGCGCGAAGCTTTTTT |
| 8_S | AAAAAATGACCAGGCCTCGAGCAAAAA | 8_A | TTTTTGCTCGAGGCCTGGTCATTTTTT |
| 9_S | AAAAAAAAGTGCCAACGTTCAAGAAAAA | 9_A | TTTTTCTTGAACGTTGGCACTTTTTTTT |
| 10_S | AAAAAAACCGCGTCGCGGCCCGGGAAAA | 10_A | TTTTCCCGGGCCGCGACGCGGTTTTTTT |
| 11_S | AAAAAAGGGCCCACTATCGACGTCAAAA | 11_A | TTTTGACGTCGATAGTGGGCCCTTTTTT |
| 12_S | AAAAAAAGCTTCTATAGCTCCATGGAAAA | 12_A | TTTTCCATGGAGCTATAGAAGCTTTTTTT |
| 13_S | AAAAAAACCCTGTGTCGGCCGATAAAAAA | 13_A | TTTTTTATCGGCCGACACAGGGTTTTTTT |
| 14_S | AAAAAAACTCAACTAGTGAACCACCAAAA | 14_A | TTTTGGTGGTTCACTAGTTGAGTTTTTTT |
| 15_S | AAAAAAATCCGATCGATGCATATATAAAA | 15_A | TTTTATATATGCATCGATCGGATTTTTTT |
| 16_S | AAAAAAGTGTTAGATTGTAGTTGATAAAA | 16_A | TTTTATCAACTACAATCTAACACTTTTTT |
| 17_S | AAAAAAGGGCGAGCGCTGGATCCACAAAA | 17_A | TTTTGTGGATCCAGCGCTCGCCCTTTTTT |
| 18_S | AAAAAATCCTGTTTGCGTTTGATTTTAAAA | 18_A | TTTTAAAATCAAACGCAAACAGGATTTTTT |
| 19_S | AAAAAATGAATCAATTTTTGGGCTTTAAAA | 19_A | TTTTAAAGCCCAAAAATTGATTCATTTTTT |
| 20_S | AAAAAAAGTTGTACTTATTATACTTTAAAA | 20_A | TTTTAAAGTATAATAAGTACAACTTTTTTT |
| 21_S | AAAAAAGGGAGTCATGCTTCTTGTTTAAAA | 21_A | TTTTAAACAAGAAGCATGACTCCCTTTTTT |
| 22_S | AAAAAATCTTCACCTCACTTCGGTTTAAAA | 22_A | TTTTAAACCGAAGTGAGGTGAAGATTTTTT |
| 23_S | AAAAAAGTTCGTGCTTAACCCAGTTTAAAA | 23_A | TTTTAAACTGGGTTAAGCACGAACTTTTTT |
| 24_S | AAAAAAAAATGCTAGCGCGTACGTAAAAAA | 24_A | TTTTTTACGTACGCGCTAGCATTTTTTTTT |
| 25_S | AAAAAAAAATCGAATTTGGACTTCCGAAAA | 25_A | TTTTCGGAAGTCCAAATTCGATTTTTTTTT |
| 26_S | AAAAAAAAATTTCGAGCCTCGGCGTAAAAA | 26_A | TTTTTACGCCGAGGCTCGAAATTTTTTTTT |
| 27_S | AAAAAAGGTTGGTACGTCGAGTTCTTAAAA | 27_A | TTTTAAGAACTCGACGTACCAACCTTTTTT |
| 28_S | AAAAAATATGTTTTCGCTTTATTCTTAAAA | 28_A | TTTTAAGAATAAAGCGAAAACATATTTTTT |
| 29_S | AAAAAAATCTTTGCGTCGAGATTCTTAAAA | 29_A | TTTTAAGAATCTCGACGCAAAGATTTTTTT |
| 30_S | AAAAAAAAGAGCCTGGGGGTTCGCATAAAA | 30_A | TTTTATGCGAACCCCCAGGCTCTTTTTTTT |
| 31_S | AAAAAATCCACGACTCAGCAGCTCTTAAAA | 31_A | TTTTAAGAGCTGCTGAGTCGTGGATTTTTT |
| 32_S | AAAAAACCTACGATGGTTCTGATCTTAAAA | 32_A | TTTTAAGATCAGAACCATCGTAGGTTTTTT |
| 33_S | AAAAAATTTTGTTTTTGCTTTTCCTTAAAA | 33_A | TTTTAAGGAAAAGCAAAAACAAAATTTTTT |
| 34_S | AAAAAAAAGGACATTTTTTCTTTTTGAAAA | 34_A | TTTTCAAAAAGAAAAAATGTCCTTTTTTTT |
| 35_S | AAAAAACCTTCCCCCTCTTCTGCCTTAAAA | 35_A | TTTTAAGGCAGAAGAGGGGGAAGGTTTTTT |
| 36_S | AAAAAATTTGGTCTTCGTCCTCGCTTAAAA | 36_A | TTTTAAGCGAGGACGAAGACCAAATTTTTT |
| 37_S | AAAAAAAAGCGTTACATCATATGGAAAAAA | 37_A | TTTTTTCCATATGATGTAACGCTTTTTTTT |
| 38_S | AAAAAATTTATACTTGATCCTGGCTTAAAA | 38_A | TTTTAAGCCAGGATCAAGTATAAATTTTTT |
| 39_S | AAAAAAAAGCTTCGGAGTCTTGAGAAAAAA | 39_A | TTTTTTCTCAAGACTCCGAAGCTTTTTTTT |
| 40_S | AAAAAACTGTCTGTCCTATATCACTTAAAA | 40_A | TTTTAAGTGATATAGGACAGACAGTTTTTT |
| 41_S | AAAAAAAACAAAACCTCCTAATAGCGAAAA | 41_A | TTTTCGCTATTAGGAGGTTTTGTTTTTTTT |
| 42_S | AAAAAATTCACTTTTACCTTTGTGTTAAAA | 42_A | TTTTAACACAAAGGTAAAAGTGAATTTTTT |
| 43_S | AAAAAAAGGTCATTGCTGCTGGTGTTAAAA | 43_A | TTTTAACACCAGCAGCAATGACCTTTTTTT |
| 44_S | AAAAAAGTACATGCGTAGATAATGTTAAAA | 44_A | TTTTAACATTATCTACGCATGTACTTTTTT |
| 45_S | AAAAAAAACGTCTGGCGGGGTTGAGAAAAA | 45_A | TTTTTCTCAACCCCGCCAGACGTTTTTTTT |
| 46_S | AAAAAACCTTACTGTTGATTCTGGTTAAAA | 46_A | TTTTAACCAGAATCAACAGTAAGGTTTTTT |
| 47_S | AAAAAAGTTGCCGACGATTGCGGGTTAAAA | 47_A | TTTTAACCCGCAATCGTCGGCAACTTTTTT |
| 48_S | AAAAAATTGCACCTGATACTAGGGTTAAAA | 48_A | TTTTAACCCTAGTATCAGGTGCAATTTTTT |
| 49_S | AAAAAATTGCATTCCACCCTTAGGTTAAAA | 49_A | TTTTAACCTAAGGGTGGAATGCAATTTTTT |
| 50_S | AAAAAATGGTCGATCCGCATGCAGTTAAAA | 50_A | TTTTAACTGCATGCGGATCGACCATTTTTT |
| 51_S | AAAAAAAACTGTCGCCCCCGGCTGGCAAAA | 51_A | TTTTGCCAGCCGGGGGCGACAGTTTTTTTT |
| 52_S | AAAAAAAACTCCCATACTACAACCGAAAAA | 52_A | TTTTTCGGTTGTAGTATGGGAGTTTTTTTT |
| 53_S | AAAAAAAATACTGCGCACTTGCACTCAAAA | 53_A | TTTTGAGTGCAAGTGCGCAGTATTTTTTTT |
| 54_S | AAAAAAGTCCGCCCTTAATTCATATTAAAA | 54_A | TTTTAATATGAATTAAGGGCGGACTTTTTT |
| 55_S | AAAAAATTCGTTTTTCGTCTTTCATTAAAA | 55_A | TTTTAATGAAAGACGAAAAACGAATTTTTT |
| 56_S | AAAAAAAATCTTAGCTCTATCTCATCAAAA | 56_A | TTTTGATGAGATAGAGCTAAGATTTTTTTT |
| 57_S | AAAAAAAATTAGTCTCTTCGTCTCCCAAAA | 57_A | TTTTGGGAGACGAAGAGACTAATTTTTTTT |
| 58_S | AAAAAACCCGGTTGGAGAAAATAATTAAAA | 58_A | TTTTAATTATTTTCTCCAACCGGGTTTTTT |
| 59_S | AAAAAAATGGTGCCGCTGAGCAAATTAAAA | 59_A | TTTTAATTTGCTCAGCGGCACCATTTTTTT |
| 60_S | AAAAAATCATTTTATTTTGCCTTTCTAAAA | 60_A | TTTTAGAAAGGCAAAATAAAATGATTTTTT |
| 61_S | AAAAAATCTTCTTGTTTCTTATTTCTAAAA | 61_A | TTTTAGAAATAAGAAACAAGAAGATTTTTT |
| 62_S | AAAAAATCTATTCCATTTCGCCTTCTAAAA | 62_A | TTTTAGAAGGCGAAATGGAATAGATTTTTT |
| 63_S | AAAAAAGCCTTTACCCTTGTTCCTCTAAAA | 63_A | TTTTAGAGGAACAAGGGTAAAGGCTTTTTT |
| 64_S | AAAAAATCGGGCCCCGTTAGCACTCTAAAA | 64_A | TTTTAGAGTGCTAACGGGGCCCGATTTTTT |
| 65_S | AAAAAAGTCATTATAACCCAAACTCTAAAA | 65_A | TTTTAGAGTTTGGGTTATAATGACTTTTTT |
| 66_S | AAAAAATATCGGGCACGGTCTTGTCTAAAA | 66_A | TTTTAGACAAGACCGTGCCCGATATTTTTT |
| 67_S | AAAAAAGGGGTCCTTTTCCAATGTCTAAAA | 67_A | TTTTAGACATTGGAAAAGGACCCCTTTTTT |
| 68_S | AAAAAATTCTTTTTTCTTCAAGGTCTAAAA | 68_A | TTTTAGACCTTGAAGAAAAAAGAATTTTTT |
| 69_S | AAAAAAAGTAGTTACTGCGAAAGTCTAAAA | 69_A | TTTTAGACTTTCGCAGTAACTACTTTTTTT |
| 70_S | AAAAAAGTGCGTTCACGCTTGTATCTAAAA | 70_A | TTTTAGATACAAGCGTGAACGCACTTTTTT |
| 71_S | AAAAAAGCTCATGGCCCTGAGGATCTAAAA | 71_A | TTTTAGATCCTCAGGGCCATGAGCTTTTTT |
| 72_S | AAAAAATATATTTTCAGCTTTAATCTAAAA | 72_A | TTTTAGATTAAAGCTGAAAATATATTTTTT |
| 73_S | AAAAAATCCCTGCTCCCCTGGTTCCTAAAA | 73_A | TTTTAGGAACCAGGGGAGCAGGGATTTTTT |
| 74_S | AAAAAACCGTCTTATCTCTCGTCCCTAAAA | 74_A | TTTTAGGGACGAGAGATAAGACGGTTTTTT |
| 75_S | AAAAAAAGGGTGCTATTCGTCGACACAAAA | 75_A | TTTTGTGTCGACGAATAGCACCCTTTTTTT |
| 76_S | AAAAAAAGGGTCCGGTGCATACATGTAAAA | 76_A | TTTTACATGTATGCACCGGACCCTTTTTTT |
| 77_S | AAAAAATTGTGCCTCTGCCCTCGCCTAAAA | 77_A | TTTTAGGCGAGGGCAGAGGCACAATTTTTT |
| 78_S | AAAAAATCCTATCTTGGCTTTGGCCTAAAA | 78_A | TTTTAGGCCAAAGCCAAGATAGGATTTTTT |
| 79_S | AAAAAAAGGTTTGGGCTTCACGTTATAAAA | 79_A | TTTTATAACGTGAAGCCCAAACCTTTTTTT |
| 80_S | AAAAAAGCTCGTCTCCGCCTCCTGCTAAAA | 80_A | TTTTAGCAGGAGGCGGAGACGAGCTTTTTT |
| 81_S | AAAAAACCGACCTGCACGTCGCTGCTAAAA | 81_A | TTTTAGCAGCGACGTGCAGGTCGGTTTTTT |
| 82_S | AAAAAATGCAACGCTCCTTTCCCGCTAAAA | 82_A | TTTTAGCGGGAAAGGAGCGTTGCATTTTTT |
| 83_S | AAAAAATATGTAGCTGCCGTTACGCTAAAA | 83_A | TTTTAGCGTAACGGCAGCTACATATTTTTT |
| 84_S | AAAAAATATTCGCCGGTAGCCACGCTAAAA | 84_A | TTTTAGCGTGGCTACCGGCGAATATTTTTT |
| 85_S | AAAAAATCGCACTGCCGCCGATGGCTAAAA | 85_A | TTTTAGCCATCGGCGGCAGTGCGATTTTTT |
| 86_S | AAAAAATTCTTACTTTTGTCTTTACTAAAA | 86_A | TTTTAGTAAAGACAAAAGTAAGAATTTTTT |
| 87_S | AAAAAACATTCCTAGTTCATGTTACTAAAA | 87_A | TTTTAGTAACATGAACTAGGAATGTTTTTT |
| 88_S | AAAAAATCAACCATTATGGTATTACTAAAA | 88_A | TTTTAGTAATACCATAATGGTTGATTTTTT |
| 89_S | AAAAAACCACCCAGTCTGTTTCTACTAAAA | 89_A | TTTTAGTAGAAACAGACTGGGTGGTTTTTT |
| 90_S | AAAAAAAGTATCAAAGCCTAGGGCTGAAAA | 90_A | TTTTCAGCCCTAGGCTTTGATACTTTTTTT |
| 91_S | AAAAAAAGTGGCCGCTAGTCAACTCAAAAA | 91_A | TTTTTGAGTTGACTAGCGGCCACTTTTTTT |
| 92_S | AAAAAAAGTCTGCGGCTGCAGGGCGGAAAA | 92_A | TTTTCCGCCCTGCAGCCGCAGACTTTTTTT |
| 93_S | AAAAAAAGTTACCAGTACGCGTCGGGAAAA | 93_A | TTTTCCCGACGCGTACTGGTAACTTTTTTT |
| 94_S | AAAAAATCAGTCTGAGACTTACAACTAAAA | 94_A | TTTTAGTTGTAAGTCTCAGACTGATTTTTT |
| 95_S | AAAAAAAGTTCGCGGATTTCGGCCATAAAA | 95_A | TTTTATGGCCGAAATCCGCGAACTTTTTTT |
| 96_S | AAAAAAAGTTCCAGGTCCGATGCTCAAAAA | 96_A | TTTTTGAGCATCGGACCTGGAACTTTTTTT |
| 97_S | AAAAAAAGTTTGACCTTGGCGTTCGTAAAA | 97_A | TTTTACGAACGCCAAGGTCAAACTTTTTTT |
| 98_S | AAAAAACGGGATTCGAAACTTTTTGTAAAA | 98_A | TTTTACAAAAAGTTTCGAATCCCGTTTTTT |
| 99_S | AAAAAAACCTCTTTATTCTCGATTGTAAAA | 99_A | TTTTACAATCGAGAATAAAGAGGTTTTTTT |
| 100_S | AAAAAAGTGTTTGTGCTTCTCTCTGTAAAA | 100_A | TTTTACAGAGAGAAGCACAAACACTTTTTT |
| 101_S | AAAAAAGTCTAAGGACTTATAGCTGTAAAA | 101_A | TTTTACAGCTATAAGTCCTTAGACTTTTTT |
| 102_S | AAAAAAACAGCTCGGTGCCACTGTGGAAAA | 102_A | TTTTCCACAGTGGCACCGAGCTGTTTTTTT |
| 103_S | AAAAAAACACCTGTATAGCATGGACGAAAA | 103_A | TTTTCGTCCATGCTATACAGGTGTTTTTTT |
| 104_S | AAAAAATGCCCTTTTTATCTTTATGTAAAA | 104_A | TTTTACATAAAGATAAAAAGGGCATTTTTT |
| 105_S | AAAAAACCGTATGTATATCTATATGTAAAA | 105_A | TTTTACATATAGATATACATACGGTTTTTT |
| 106_S | AAAAAAACATCCACACCCGCGGCGCGAAAA | 106_A | TTTTCGCGCCGCGGGTGTGGATGTTTTTTT |
| 107_S | AAAAAAGTTTCTGCTTTCCCTTTCGTAAAA | 107_A | TTTTACGAAAGGGAAAGCAGAAACTTTTTT |
| 108_S | AAAAAAGGCGTCTGACTGTAACTCGTAAAA | 108_A | TTTTACGAGTTACAGTCAGACGCCTTTTTT |
| 109_S | AAAAAATACGTCTACGTGGTTGTCGTAAAA | 109_A | TTTTACGACAACCACGTAGACGTATTTTTT |
| 110_S | AAAAAAGCGTAAGCCTTGACAGTCGTAAAA | 110_A | TTTTACGACTGTCAAGGCTTACGCTTTTTT |
| 111_S | AAAAAACCCATCCTTCGTTCCATCGTAAAA | 111_A | TTTTACGATGGAACGAAGGATGGGTTTTTT |
| 112_S | AAAAAACTTCTAGTTTATGCTTCCGTAAAA | 112_A | TTTTACGGAAGCATAAACTAGAAGTTTTTT |
| 113_S | AAAAAAACTTCTGTCCCTGTTCCCGTAAAA | 113_A | TTTTACGGGAACAGGGACAGAAGTTTTTTT |
| 114_S | AAAAAAACGGGTTGTGAATGTGGAAGAAAA | 114_A | TTTTCTTCCACATTCACAACCCGTTTTTTT |
| 115_S | AAAAAATCGTAAATGCCCATAGCCGTAAAA | 115_A | TTTTACGGCTATGGGCATTTACGATTTTTT |
| 116_S | AAAAAAACGGTGTCATAGTATTAGTGAAAA | 116_A | TTTTCACTAATACTATGACACCGTTTTTTT |
| 117_S | AAAAAAATCAGCCTGACTCTGGGCGTAAAA | 117_A | TTTTACGCCCAGAGTCAGGCTGATTTTTTT |
| 118_S | AAAAAAACGCTATATATTGGTGCTAGAAAA | 118_A | TTTTCTAGCACCAATATATAGCGTTTTTTT |
| 119_S | AAAAAAACGCTCAAGTCTATCAGTAGAAAA | 119_A | TTTTCTACTGATAGACTTGAGCGTTTTTTT |
| 120_S | AAAAAATTCCGCGTGTATGTGTACGTAAAA | 120_A | TTTTACGTACACATACACGCGGAATTTTTT |
| 121_S | AAAAAAGCTAGTGCTGCCACCAACGTAAAA | 121_A | TTTTACGTTGGTGGCAGCACTAGCTTTTTT |
| 122_S | AAAAAATACTCTTCCTCGTTCTTGGTAAAA | 122_A | TTTTACCAAGAACGAGGAAGAGTATTTTTT |
| 123_S | AAAAAAGGAAAACGAGTTCATCTGGTAAAA | 123_A | TTTTACCAGATGAACTCGTTTTCCTTTTTT |
| 124_S | AAAAAAACTCACGGCGTCACTATGGTAAAA | 124_A | TTTTACCATAGTGACGCCGTGAGTTTTTTT |
| 125_S | AAAAAACACAGCATTAGGCTATCGGTAAAA | 125_A | TTTTACCGATAGCCTAATGCTGTGTTTTTT |
| 126_S | AAAAAAACCGATTACCATTTTTACTGAAAA | 126_A | TTTTCAGTAAAAATGGTAATCGGTTTTTTT |
| 127_S | AAAAAATTAGACGCTACACTTACGGTAAAA | 127_A | TTTTACCGTAAGTGTAGCGTCTAATTTTTT |
| 128_S | AAAAAAACCCGGCCGCCATGGTAGTAAAAA | 128_A | TTTTTACTACCATGGCGGCCGGGTTTTTTT |
| 129_S | AAAAAAACCCGTGCCTGCCTATTGCAAAAA | 129_A | TTTTTGCAATAGGCAGGCACGGGTTTTTTT |
| 130_S | AAAAAACGCAACTCCGGATGTGGGGTAAAA | 130_A | TTTTACCCCACATCCGGAGTTGCGTTTTTT |
| 131_S | AAAAAACGTTAACACTGATTTAGGGTAAAA | 131_A | TTTTACCCTAAATCAGTGTTAACGTTTTTT |
| 132_S | AAAAAAGGTAGTCACATTACGAGGGTAAAA | 132_A | TTTTACCCTCGTAATGTGACTACCTTTTTT |
| 133_S | AAAAAATAAACGTGTTCATACTTAGTAAAA | 133_A | TTTTACTAAGTATGAACACGTTTATTTTTT |
| 134_S | AAAAAAACTATAGTCTTTGTCATGATAAAA | 134_A | TTTTATCATGACAAAGACTATAGTTTTTTT |
| 135_S | AAAAAATAGCGACTTCGACTCTCAGTAAAA | 135_A | TTTTACTGAGAGTCGAAGTCGCTATTTTTT |
| 136_S | AAAAAATAGTGTGATTGGTATCCAGTAAAA | 136_A | TTTTACTGGATACCAATCACACTATTTTTT |
| 137_S | AAAAAAACTCTAGGTCAATATTATTCAAAA | 137_A | TTTTGAATAATATTGACCTAGAGTTTTTTT |
| 138_S | AAAAAAACTCTCCGGGTACAATTGCAAAAA | 138_A | TTTTTGCAATTGTACCCGGAGAGTTTTTTT |
| 139_S | AAAAAAACTTTCTATTCTGGATAAGTAAAA | 139_A | TTTTACTTATCCAGAATAGAAAGTTTTTTT |
| 140_S | AAAAAAATCGTTGTTATCGTATTTATAAAA | 140_A | TTTTATAAATACGATAACAACGATTTTTTT |
| 141_S | AAAAAATCTTACCCCTCCGCTTCTATAAAA | 141_A | TTTTATAGAAGCGGAGGGGTAAGATTTTTT |
| 142_S | AAAAAACCAACTCTCTTTCGGACTATAAAA | 142_A | TTTTATAGTCCGAAAGAGAGTTGGTTTTTT |
| 143_S | AAAAAATGTCCATCTTGCGTCTGTATAAAA | 143_A | TTTTATACAGACGCAAGATGGACATTTTTT |
| 144_S | AAAAAAGTACGGATGCCCGCGCGTATAAAA | 144_A | TTTTATACGCGCGGGCATCCGTACTTTTTT |
| 145_S | AAAAAAGGTAATGCCGGTCCCAGTATAAAA | 145_A | TTTTATACTGGGACCGGCATTACCTTTTTT |
| 146_S | AAAAAATATCCTATTCTAATTTATATAAAA | 146_A | TTTTATATAAATTAGAATAGGATATTTTTT |
| 147_S | AAAAAAATATTTGAGCTAGGTTATCAAAAA | 147_A | TTTTTGATAACCTAGCTCAAATATTTTTTT |
| 148_S | AAAAAATCACAATACCTTCTCGTCATAAAA | 148_A | TTTTATGACGAGAAGGTATTGTGATTTTTT |
| 149_S | AAAAAAGAAATACCAGAAACTATCATAAAA | 149_A | TTTTATGATAGTTTCTGGTATTTCTTTTTT |
| 150_S | AAAAAAATGATTGCCAATGCTCGCAAAAAA | 150_A | TTTTTTGCGAGCATTGGCAATCATTTTTTT |
| 151_S | AAAAAAATGATTTGCAGATCTCCGAGAAAA | 151_A | TTTTCTCGGAGATCTGCAAATCATTTTTTT |
| 152_S | AAAAAACTGCTTGCCCCTGTCTCCATAAAA | 152_A | TTTTATGGAGACAGGGGCAAGCAGTTTTTT |
| 153_S | AAAAAATGTATTCGTGTCCACTCCATAAAA | 153_A | TTTTATGGAGTGGACACGAATACATTTTTT |
| 154_S | AAAAAAATCTCACTCCTGGTCCCCATAAAA | 154_A | TTTTATGGGGACCAGGAGTGAGATTTTTTT |
| 155_S | AAAAAAATGCGCCCTTTCTGACATAAAAAA | 155_A | TTTTTTATGTCAGAAAGGGCGCATTTTTTT |
| 156_S | AAAAAATTTGTACTTTAAGTTTACATAAAA | 156_A | TTTTATGTAAACTTAAAGTACAAATTTTTT |
| 157_S | AAAAAAATGTTCAAGCTCGATCTACAAAAA | 157_A | TTTTTGTAGATCGAGCTTGAACATTTTTTT |
| 158_S | AAAAAAATCACGGTTCGACCTCCAGCAAAA | 158_A | TTTTGCTGGAGGTCGAACCGTGATTTTTTT |
| 159_S | AAAAAAATCATCCTATTGTTGCTGTCAAAA | 159_A | TTTTGACAGCAACAATAGGATGATTTTTTT |
| 160_S | AAAAAACGGTCGCGATCGTTAATGATAAAA | 160_A | TTTTATCATTAACGATCGCGACCGTTTTTT |
| 161_S | AAAAAACTGCTAATCGGTGACCCGATAAAA | 161_A | TTTTATCGGGTCACCGATTAGCAGTTTTTT |
| 162_S | AAAAAAGTCCATAAACTAAATTGGATAAAA | 162_A | TTTTATCCAATTTAGTTTATGGACTTTTTT |
| 163_S | AAAAAAATCCATATTATTGCTACCCGAAAA | 163_A | TTTTCGGGTAGCAATAATATGGATTTTTTT |
| 164_S | AAAAAAGTCTCACCTTTAGTCTTAATAAAA | 164_A | TTTTATTAAGACTAAAGGTGAGACTTTTTT |
| 165_S | AAAAAAATTGTATCATACTTCAGTTGAAAA | 165_A | TTTTCAACTGAAGTATGATACAATTTTTTT |
| 166_S | AAAAAATATTCCGCTTTTGGCTGAATAAAA | 166_A | TTTTATTCAGCCAAAAGCGGAATATTTTTT |
| 167_S | AAAAAAATTCATGCTATTTAACCTTAAAAA | 167_A | TTTTTAAGGTTAAATAGCATGAATTTTTTT |
| 168_S | AAAAAAATTCGCAACCCTCACGTTCGAAAA | 168_A | TTTTCGAACGTGAGGGTTGCGAATTTTTTT |
| 169_S | AAAAAAGACCGCAGCAAGAATTAAATAAAA | 169_A | TTTTATTTAATTCTTGCTGCGGTCTTTTTT |
| 170_S | AAAAAATGGAAGGAGAGGGATGAAATAAAA | 170_A | TTTTATTTCATCCCTCTCCTTCCATTTTTT |
| 171_S | AAAAAAATTTTTAGTTTTAATTTTGGAAAA | 171_A | TTTTCCAAAATTAAAACTAAAAATTTTTTT |
| 172_S | AAAAAATCCTTTTCTCTTTCTGTTTCAAAA | 172_A | TTTTGAAACAGAAAGAGAAAAGGATTTTTT |
| 173_S | AAAAAATACATTTGACTTTGGGTTTCAAAA | 173_A | TTTTGAAACCCAAAGTCAAATGTATTTTTT |
| 174_S | AAAAAATTTCTCCTTCTTTGTTCTTCAAAA | 174_A | TTTTGAAGAACAAAGAAGGAGAAATTTTTT |
| 175_S | AAAAAATTAGTCCTGATTCAATCTTCAAAA | 175_A | TTTTGAAGATTGAATCAGGACTAATTTTTT |
| 176_S | AAAAAATCTGTTCTCGTTTCCCCTTCAAAA | 176_A | TTTTGAAGGGGAAACGAGAACAGATTTTTT |
| 177_S | AAAAAATAAATCGCGGCTACTGCTTCAAAA | 177_A | TTTTGAAGCAGTAGCCGCGATTTATTTTTT |
| 178_S | AAAAAATTGGTTCTCATTTCTACTTCAAAA | 178_A | TTTTGAAGTAGAAATGAGAACCAATTTTTT |
| 179_S | AAAAAACCCCAGATTTCCAGCAGTTCAAAA | 179_A | TTTTGAACTGCTGGAAATCTGGGGTTTTTT |
| 180_S | AAAAAACACTGCACTATTGGGAGTTCAAAA | 180_A | TTTTGAACTCCCAATAGTGCAGTGTTTTTT |
| 181_S | AAAAAACCGGCACGATTTGGATATTCAAAA | 181_A | TTTTGAATATCCAAATCGTGCCGGTTTTTT |
| 182_S | AAAAAAGTCTGCGTCCTACCCCATTCAAAA | 182_A | TTTTGAATGGGGTAGGACGCAGACTTTTTT |
| 183_S | AAAAAAGAGAATTGTCAGTGCCCAACAAAA | 183_A | TTTTGTTGGGCACTGACAATTCTCTTTTTT |
| 184_S | AAAAAATCCATGTTTAAGCTGGTCTCAAAA | 184_A | TTTTGAGACCAGCTTAAACATGGATTTTTT |
| 185_S | AAAAAACTGTGTCCCCGTCCCATCTCAAAA | 185_A | TTTTGAGATGGGACGGGGACACAGTTTTTT |
| 186_S | AAAAAATTCTTAGTTAGATTCACCTCAAAA | 186_A | TTTTGAGGTGAATCTAACTAAGAATTTTTT |
| 187_S | AAAAAACGCCGGACAATCAGGCGCTCAAAA | 187_A | TTTTGAGCGCCTGATTGTCCGGCGTTTTTT |
| 188_S | AAAAAACACTCACACCTATACGGCTCAAAA | 188_A | TTTTGAGCCGTATAGGTGTGAGTGTTTTTT |
| 189_S | AAAAAACTTGCTCCTCCGTTTTACTCAAAA | 189_A | TTTTGAGTAAAACGGAGGAGCAAGTTTTTT |
| 190_S | AAAAAAGAGTGGTTTATTAATGTATAAAAA | 190_A | TTTTTATACATTAATAAACCACTCTTTTTT |
| 191_S | AAAAAATCGGTACGCACCCCGGACTCAAAA | 191_A | TTTTGAGTCCGGGGTGCGTACCGATTTTTT |
| 192_S | AAAAAATAAGTAACCGAACGAAACTCAAAA | 192_A | TTTTGAGTTTCGTTCGGTTACTTATTTTTT |
| 193_S | AAAAAAGACACTCTAATTCCGTAGGCAAAA | 193_A | TTTTGCCTACGGAATTAGAGTGTCTTTTTT |
| 194_S | AAAAAATCCGTTCCCATTTGTCCGTCAAAA | 194_A | TTTTGACGGACAAATGGGAACGGATTTTTT |
| 195_S | AAAAAATGGTCGTCGTACTCGTGGTCAAAA | 195_A | TTTTGACCACGAGTACGACGACCATTTTTT |
| 196_S | AAAAAACTACAACCTGCGCGGTGGTCAAAA | 196_A | TTTTGACCACCGCGCAGGTTGTAGTTTTTT |
| 197_S | AAAAAATGGAAAGACCTATACCGGTCAAAA | 197_A | TTTTGACCGGTATAGGTCTTTCCATTTTTT |
| 198_S | AAAAAATATCTGATCCAATTTAGGTCAAAA | 198_A | TTTTGACCTAAATTGGATCAGATATTTTTT |
| 199_S | AAAAAAGATGATTAGTAATTGCGGAAAAAA | 199_A | TTTTTTCCGCAATTACTAATCATCTTTTTT |
| 200_S | AAAAAATATGGCACTCGCCGCACATCAAAA | 200_A | TTTTGATGTGCGGCGAGTGCCATATTTTTT |
| 201_S | AAAAAAGATCTAGGCCTTGGGACTCAAAAA | 201_A | TTTTTGAGTCCCAAGGCCTAGATCTTTTTT |
| 202_S | AAAAAAGATTGACTCGTCTTACTCGAAAAA | 202_A | TTTTTCGAGTAAGACGAGTCAATCTTTTTT |
| 203_S | AAAAAACTCCCTCTCCTCTGTCTTCCAAAA | 203_A | TTTTGGAAGACAGAGGAGAGGGAGTTTTTT |
| 204_S | AAAAAAGGAAGTACTGACGATCTGGAAAAA | 204_A | TTTTTCCAGATCGTCAGTACTTCCTTTTTT |
| 205_S | AAAAAATTCCTGTCCGCTCTCGCTCCAAAA | 205_A | TTTTGGAGCGAGAGCGGACAGGAATTTTTT |
| 206_S | AAAAAATTCTAACTTAGCCTATGTCCAAAA | 206_A | TTTTGGACATAGGCTAAGTTAGAATTTTTT |
| 207_S | AAAAAAGGACGTCTTCGGTATGAGTGAAAA | 207_A | TTTTCACTCATACCGAAGACGTCCTTTTTT |
| 208_S | AAAAAAGCTGTCACTGTAGTCGGTCCAAAA | 208_A | TTTTGGACCGACTACAGTGACAGCTTTTTT |
| 209_S | AAAAAATCCATTCTTCGCGTTTATCCAAAA | 209_A | TTTTGGATAAACGCGAAGAATGGATTTTTT |
| 210_S | AAAAAAGTGTGTGGCTCAGGATTCCCAAAA | 210_A | TTTTGGGAATCCTGAGCCACACACTTTTTT |
| 211_S | AAAAAACCTCTTGTCTCTGCTCTCCCAAAA | 211_A | TTTTGGGAGAGCAGAGACAAGAGGTTTTTT |
| 212_S | AAAAAACCTTACCTCATCCCTATCCCAAAA | 212_A | TTTTGGGATAGGGATGAGGTAAGGTTTTTT |
| 213_S | AAAAAACTTCCCTCCTTGTCCTCCCCAAAA | 213_A | TTTTGGGGAGGACAAGGAGGGAAGTTTTTT |
| 214_S | AAAAAATTTGCGCTTCATCCTGCCCCAAAA | 214_A | TTTTGGGGCAGGATGAAGCGCAAATTTTTT |
| 215_S | AAAAAATTTCTATTTCATCTTTGCCCAAAA | 215_A | TTTTGGGCAAAGATGAAATAGAAATTTTTT |
| 216_S | AAAAAATAATATCGATACTCATGCCCAAAA | 216_A | TTTTGGGCATGAGTATCGATATTATTTTTT |
| 217_S | AAAAAAGGGTATACTCGCAGCCCCTAAAAA | 217_A | TTTTTAGGGGCTGCGAGTATACCCTTTTTT |
| 218_S | AAAAAAGGGTCGCTGTGAGCTCATGAAAAA | 218_A | TTTTTCATGAGCTCACAGCGACCCTTTTTT |
| 219_S | AAAAAAGGGTTTAGATGTCCTTCAAAAAAA | 219_A | TTTTTTTGAAGGACATCTAAACCCTTTTTT |
| 220_S | AAAAAAGGGTTTGTCGGGAATTGCCGAAAA | 220_A | TTTTCGGCAATTCCCGACAAACCCTTTTTT |
| 221_S | AAAAAAGCATATCTTCAGAACTTGCCAAAA | 221_A | TTTTGGCAAGTTCTGAAGATATGCTTTTTT |
| 222_S | AAAAAAGGCGGTCGTAGTTGTGGGAGAAAA | 222_A | TTTTCTCCCACAACTACGACCGCCTTTTTT |
| 223_S | AAAAAAGGCCACGGTAACCCCTTTTGAAAA | 223_A | TTTTCAAAAGGGGTTACCGTGGCCTTTTTT |
| 224_S | AAAAAAGGCCGTTGGACCTCGGTCTAAAAA | 224_A | TTTTTAGACCGAGGTCCAACGGCCTTTTTT |
| 225_S | AAAAAAGGCCTTCGCTCGTGTTTACGAAAA | 225_A | TTTTCGTAAACACGAGCGAAGGCCTTTTTT |
| 226_S | AAAAAAGGCTGTGGCCAATGTTCGTAAAAA | 226_A | TTTTTACGAACATTGGCCACAGCCTTTTTT |
| 227_S | AAAAAAGGCTCCGGCCCCCAATCGCAAAAA | 227_A | TTTTTGCGATTGGGGGCCGGAGCCTTTTTT |
| 228_S | AAAAAAGGCTTCCACTTTGAGTTTGCAAAA | 228_A | TTTTGCAAACTCAAAGTGGAAGCCTTTTTT |
| 229_S | AAAAAAGCCCCTTGCGCCTCTCTACCAAAA | 229_A | TTTTGGTAGAGAGGCGCAAGGGGCTTTTTT |
| 230_S | AAAAAAGGTGAGGAAGTTGGGTATGGAAAA | 230_A | TTTTCCATACCCAACTTCCTCACCTTTTTT |
| 231_S | AAAAAATTTTGTGGTGTTTCGCCACCAAAA | 231_A | TTTTGGTGGCGAAACACCACAAAATTTTTT |
| 232_S | AAAAAATAGGCGTTATGGCTAACACCAAAA | 232_A | TTTTGGTGTTAGCCATAACGCCTATTTTTT |
| 233_S | AAAAAAGGTCCAGTCCACGTCATCAGAAAA | 233_A | TTTTCTGATGACGTGGACTGGACCTTTTTT |
| 234_S | AAAAAAGGTCTGGACGATCACCGGGAAAAA | 234_A | TTTTTCCCGGTGATCGTCCAGACCTTTTTT |
| 235_S | AAAAAAGGTTTAGCCCATGCCTGTGCAAAA | 235_A | TTTTGCACAGGCATGGGCTAAACCTTTTTT |
| 236_S | AAAAAATTACTTCTCCCTTCCTTTGCAAAA | 236_A | TTTTGCAAAGGAAGGGAGAAGTAATTTTTT |
| 237_S | AAAAAATCCCCCCGTTTCACTCTTGCAAAA | 237_A | TTTTGCAAGAGTGAAACGGGGGGATTTTTT |
| 238_S | AAAAAATCGGGGATCCGGGCGGTTGCAAAA | 238_A | TTTTGCAACCGCCCGGATCCCCGATTTTTT |
| 239_S | AAAAAAGTCGTTACACGTATGCGTGCAAAA | 239_A | TTTTGCACGCATACGTGTAACGACTTTTTT |
| 240_S | AAAAAATTCGTATCTGTACCCTATGCAAAA | 240_A | TTTTGCATAGGGTACAGATACGAATTTTTT |
| 241_S | AAAAAAGTTATTATACCTGATCTCGCAAAA | 241_A | TTTTGCGAGATCAGGTATAATAACTTTTTT |
| 242_S | AAAAAACAGTTCGATGTCACCCCCGCAAAA | 242_A | TTTTGCGGGGGTGACATCGAACTGTTTTTT |
| 243_S | AAAAAAGCGGTAGATGCGCAACTGTGAAAA | 243_A | TTTTCACAGTTGCGCATCTACCGCTTTTTT |
| 244_S | AAAAAAGCGCGAATTCTAGAACTATAAAAA | 244_A | TTTTTATAGTTCTAGAATTCGCGCTTTTTT |
| 245_S | AAAAAAGCGCGCCCGCACTGTTAATAAAAA | 245_A | TTTTTATTAACAGTGCGGGCGCGCTTTTTT |
| 246_S | AAAAAACTGACCGTCAGCGCCCACGCAAAA | 246_A | TTTTGCGTGGGCGCTGACGGTCAGTTTTTT |
| 247_S | AAAAAATCTAGGGGTACCTATTTGGCAAAA | 247_A | TTTTGCCAAATAGGTACCCCTAGATTTTTT |
| 248_S | AAAAAAGCCATCATGGTTAGTGTACAAAAA | 248_A | TTTTTGTACACTAACCATGATGGCTTTTTT |
| 249_S | AAAAAATGCCGAGACCCCATGCCGGCAAAA | 249_A | TTTTGCCGGCATGGGGTCTCGGCATTTTTT |
| 250_S | AAAAAAGCCGTGGGTGATGGTCACAGAAAA | 250_A | TTTTCTGTGACCATCACCCACGGCTTTTTT |
| 251_S | AAAAAACATTGATAAAATCTCCAGGCAAAA | 251_A | TTTTGCCTGGAGATTTTATCAATGTTTTTT |
| 252_S | AAAAAATTCTAGCTTTTAACTTTAGCAAAA | 252_A | TTTTGCTAAAGTTAAAAGCTAGAATTTTTT |
| 253_S | AAAAAAGCTAGTCGATTCGGCTTAGAAAAA | 253_A | TTTTTCTAAGCCGAATCGACTAGCTTTTTT |
| 254_S | AAAAAACAACTTGGAGGATTTACAGCAAAA | 254_A | TTTTGCTGTAAATCCTCCAAGTTGTTTTTT |
| 255_S | AAAAAAGCTCCCGTCTCTTAGTTCTAAAAA | 255_A | TTTTTAGAACTAAGAGACGGGAGCTTTTTT |
| 256_S | AAAAAAGCTTCTCATGTTGTCTTAGGAAAA | 256_A | TTTTCCTAAGACAACATGAGAAGCTTTTTT |
| 257_S | AAAAAAGCTTTCAACATCTGCGTCCAAAAA | 257_A | TTTTTGGACGCAGATGTTGAAAGCTTTTTT |
| 258_S | AAAAAACATAACCGCAAAATCCCTACAAAA | 258_A | TTTTGTAGGGATTTTGCGGTTATGTTTTTT |
| 259_S | AAAAAAGTAGGTGCTGATGCTGGCCGAAAA | 259_A | TTTTCGGCCAGCATCAGCACCTACTTTTTT |
| 260_S | AAAAAAGTATAACGTGTTGTTGACCGAAAA | 260_A | TTTTCGGTCAACAACACGTTATACTTTTTT |
| 261_S | AAAAAAGTCGTCACGCACGTGAATACAAAA | 261_A | TTTTGTATTCACGTGCGTGACGACTTTTTT |
| 262_S | AAAAAATTTGCTCTTTTGGTTTTCACAAAA | 262_A | TTTTGTGAAAACCAAAAGAGCAAATTTTTT |
| 263_S | AAAAAACCCGCCGTCGTGATTATCACAAAA | 263_A | TTTTGTGATAATCACGACGGCGGGTTTTTT |
| 264_S | AAAAAATAATTACAAGTCGGCTCCACAAAA | 264_A | TTTTGTGGAGCCGACTTGTAATTATTTTTT |
| 265_S | AAAAAAGTGCACGACCCATTTATATAAAAA | 265_A | TTTTTATATAAATGGGTCGTGCACTTTTTT |
| 266_S | AAAAAATAGTACCGCTAATGGCTGACAAAA | 266_A | TTTTGTCAGCCATTAGCGGTACTATTTTTT |
| 267_S | AAAAAACTCTTAAGGAAAGTTCCGACAAAA | 267_A | TTTTGTCGGAACTTTCCTTAAGAGTTTTTT |
| 268_S | AAAAAAGTCCCGCGCTAAGCTTTAAAAAAA | 268_A | TTTTTTTAAAGCTTAGCGCGGGACTTTTTT |
| 269_S | AAAAAATGGTACTCAGAGTTATAGACAAAA | 269_A | TTTTGTCTATAACTCTGAGTACCATTTTTT |
| 270_S | AAAAAACATGTCGCACCTGTACTAACAAAA | 270_A | TTTTGTTAGTACAGGTGCGACATGTTTTTT |
| 271_S | AAAAAACGTTGAACCTGGGCCTCAACAAAA | 271_A | TTTTGTTGAGGCCCAGGTTCAACGTTTTTT |
| 272_S | AAAAAAGTTCGGGGCTTGGTTTGGAAAAAA | 272_A | TTTTTTCCAAACCAAGCCCCGAACTTTTTT |
| 273_S | AAAAAATGATGTTGGCGCATTTAAACAAAA | 273_A | TTTTGTTTAAATGCGCCAACATCATTTTTT |
| 274_S | AAAAAAGTTTCCTACTTGTGTCTGTGAAAA | 274_A | TTTTCACAGACACAAGTAGGAAACTTTTTT |
| 275_S | AAAAAATCATCTCTATGTTATTGTTGAAAA | 275_A | TTTTCAACAATAACATAGAGATGATTTTTT |
| 276_S | AAAAAACATCGCTGTCGTTGACGTTGAAAA | 276_A | TTTTCAACGTCAACGACAGCGATGTTTTTT |
| 277_S | AAAAAATGGCTCGGGTTCCAAGGTTGAAAA | 277_A | TTTTCAACCTTGGAACCCGAGCCATTTTTT |
| 278_S | AAAAAATAGACCCTGACCCACCATTGAAAA | 278_A | TTTTCAATGGTGGGTCAGGGTCTATTTTTT |
| 279_S | AAAAAACACTTAACTGAGTGGGATTGAAAA | 279_A | TTTTCAATCCCACTCAGTTAAGTGTTTTTT |
| 280_S | AAAAAATTAATCCATGCTTATGTCTGAAAA | 280_A | TTTTCAGACATAAGCATGGATTAATTTTTT |
| 281_S | AAAAAACCCGTTCTACCTTATTCCTGAAAA | 281_A | TTTTCAGGAATAAGGTAGAACGGGTTTTTT |
| 282_S | AAAAAACAGGCCGATTCCGATCCCGGAAAA | 282_A | TTTTCCGGGATCGGAATCGGCCTGTTTTTT |
| 283_S | AAAAAACCGATCATCACATATCACTGAAAA | 283_A | TTTTCAGTGATATGTGATGATCGGTTTTTT |
| 284_S | AAAAAATTTGTTCGGGCTCATAACTGAAAA | 284_A | TTTTCAGTTATGAGCCCGAACAAATTTTTT |
| 285_S | AAAAAATTAGCTTATCCTTGCTTGTGAAAA | 285_A | TTTTCACAAGCAAGGATAAGCTAATTTTTT |
| 286_S | AAAAAACTAACCGTCGCAGGCGTGTGAAAA | 286_A | TTTTCACACGCCTGCGACGGTTAGTTTTTT |
| 287_S | AAAAAACACGCCATTGACACTGGTAAAAAA | 287_A | TTTTTTACCAGTGTCAATGGCGTGTTTTTT |
| 288_S | AAAAAATATGCTACTCCACCTTGATGAAAA | 288_A | TTTTCATCAAGGTGGAGTAGCATATTTTTT |
| 289_S | AAAAAATGCCATTGTACCTGTGTTCGAAAA | 289_A | TTTTCGAACACAGGTACAATGGCATTTTTT |
| 290_S | AAAAAATGTCTAGACTCGGACATTCGAAAA | 290_A | TTTTCGAATGTCCGAGTCTAGACATTTTTT |
| 291_S | AAAAAACGCCTTATGCCTCCCGCTCGAAAA | 291_A | TTTTCGAGCGGGAGGCATAAGGCGTTTTTT |
| 292_S | AAAAAACCGCTTACATTGCGTTGTCGAAAA | 292_A | TTTTCGACAACGCAATGTAAGCGGTTTTTT |
| 293_S | AAAAAATGTCGGCTGTTAGGATATCGAAAA | 293_A | TTTTCGATATCCTAACAGCCGACATTTTTT |
| 294_S | AAAAAACGTGCGCGTGCATGTCATCGAAAA | 294_A | TTTTCGATGACATGCACGCGCACGTTTTTT |
| 295_S | AAAAAACGGAAGATCGTGACTGCTAGAAAA | 295_A | TTTTCTAGCAGTCACGATCTTCCGTTTTTT |
| 296_S | AAAAAATTCAGAGCTTCGAGGTTCCGAAAA | 296_A | TTTTCGGAACCTCGAAGCTCTGAATTTTTT |
| 297_S | AAAAAATGGTTTCAATTTTCCGTCCGAAAA | 297_A | TTTTCGGACGGAAAATTGAAACCATTTTTT |
| 298_S | AAAAAATTCCCAGCGTATCCGACCCGAAAA | 298_A | TTTTCGGGTCGGATACGCTGGGAATTTTTT |
| 299_S | AAAAAACTAGGTATCGGTAGGCGCCGAAAA | 299_A | TTTTCGGCGCCTACCGATACCTAGTTTTTT |
| 300_S | AAAAAACTCGCGCTTGCATCTGTGCGAAAA | 300_A | TTTTCGCACAGATGCAAGCGCGAGTTTTTT |
| 301_S | AAAAAACGCATCATAGCTACGCCCCAAAAA | 301_A | TTTTTGGGGCGTAGCTATGATGCGTTTTTT |
| 302_S | AAAAAACGCTCCGTTTTTGCAATGCGAAAA | 302_A | TTTTCGCATTGCAAAAACGGAGCGTTTTTT |
| 303_S | AAAAAATGTGCCCGTGTGTGCTCGCGAAAA | 303_A | TTTTCGCGAGCACACACGGGCACATTTTTT |
| 304_S | AAAAAACTACTACCTTTGCCTAAGCGAAAA | 304_A | TTTTCGCTTAGGCAAAGGTAGTAGTTTTTT |
| 305_S | AAAAAACGCTTTCAGTTTTGATCTTAAAAA | 305_A | TTTTTAAGATCAAAACTGAAAGCGTTTTTT |
| 306_S | AAAAAACTCGTACATCTATCGCTACGAAAA | 306_A | TTTTCGTAGCGATAGATGTACGAGTTTTTT |
| 307_S | AAAAAATGCCCATGTGCACCCGTACGAAAA | 307_A | TTTTCGTACGGGTGCACATGGGCATTTTTT |
| 308_S | AAAAAACGTATTGATGTCTATTTCAAAAAA | 308_A | TTTTTTGAAATAGACATCAATACGTTTTTT |
| 309_S | AAAAAACCAGCCTCAGTCCTGAACAAAAAA | 309_A | TTTTTTGTTCAGGACTGAGGCTGGTTTTTT |
| 310_S | AAAAAACCAGTCTACACCGCTGTAGGAAAA | 310_A | TTTTCCTACAGCGGTGTAGACTGGTTTTTT |
| 311_S | AAAAAACCCTAACGAATGCTTTTCGGAAAA | 311_A | TTTTCCGAAAAGCATTCGTTAGGGTTTTTT |
| 312_S | AAAAAACCGAGTGTTGCCAGTTGCAGAAAA | 312_A | TTTTCTGCAACTGGCAACACTCGGTTTTTT |
| 313_S | AAAAAATCGCGAACGTACGTCCGCGGAAAA | 313_A | TTTTCCGCGGACGTACGTTCGCGATTTTTT |
| 314_S | AAAAAATTGGCACACATGATCTCGGGAAAA | 314_A | TTTTCCCGAGATCATGTGTGCCAATTTTTT |
| 315_S | AAAAAATTCAAAATGATCACTACGGGAAAA | 315_A | TTTTCCCGTAGTGATCATTTTGAATTTTTT |
| 316_S | AAAAAATTACTATTACACACTTGGGGAAAA | 316_A | TTTTCCCCAAGTGTGTAATAGTAATTTTTT |
| 317_S | AAAAAACCCTACATCGCATATAAGAGAAAA | 317_A | TTTTCTCTTATATGCGATGTAGGGTTTTTT |
| 318_S | AAAAAATTAGGACCATCCCCACTAGGAAAA | 318_A | TTTTCCTAGTGGGGATGGTCCTAATTTTTT |
| 319_S | AAAAAATGTCTTCTTACACCCTCTAGAAAA | 319_A | TTTTCTAGAGGGTGTAAGAAGACATTTTTT |
| 320_S | AAAAAACTACGTATATTCAGGCCCTAAAAA | 320_A | TTTTTAGGGCCTGAATATACGTAGTTTTTT |
| 321_S | AAAAAACTGGCGACTGCCAGTGGTAGAAAA | 321_A | TTTTCTACCACTGGCAGTCGCCAGTTTTTT |
| 322_S | AAAAAACTACCTCTACTCTAGCCTTAAAAA | 322_A | TTTTTAAGGCTAGAGTAGAGGTAGTTTTTT |
| 323_S | AAAAAATGAGAGAATTTCCGGTTCAGAAAA | 323_A | TTTTCTGAACCGGAAATTCTCTCATTTTTT |
| 324_S | AAAAAACTGGATGCGCTCACCAGTTAAAAA | 324_A | TTTTTAACTGGTGAGCGCATCCAGTTTTTT |
| 325_S | AAAAAACTGGTGTATCTCCTCAATTAAAAA | 325_A | TTTTTAATTGAGGAGATACACCAGTTTTTT |
| 326_S | AAAAAACTCGACATAGAATGCGGAAAAAAA | 326_A | TTTTTTTCCGCATTCTATGTCGAGTTTTTT |
| 327_S | AAAAAATCAAAACAACGATGCCGTTAAAAA | 327_A | TTTTTAACGGCATCGTTGTTTTGATTTTTT |
| 328_S | AAAAAATGTTGTTGCTGTTGGTCCTAAAAA | 328_A | TTTTTAGGACCAACAGCAACAACATTTTTT |
| 329_S | AAAAAATATCATTGTTACCGTTGCTAAAAA | 329_A | TTTTTAGCAACGGTAACAATGATATTTTTT |
| 330_S | AAAAAATGGGGCTCTGGCCCACCGTAAAAA | 330_A | TTTTTACGGTGGGCCAGAGCCCCATTTTTT |
| 331_S | AAAAAATTGCCGTCATAATTGTGGTAAAAA | 331_A | TTTTTACCACAATTATGACGGCAATTTTTT |
| 332_S | AAAAAATGCAGACCTTCAGCTGGGTAAAAA | 332_A | TTTTTACCCAGCTGAAGGTCTGCATTTTTT |
| 333_S | AAAAAATACTGGCTGTCTCGTTCACAAAAA | 333_A | TTTTTGTGAACGAGACAGCCAGTATTTTTT |
| 334_S | AAAAAATTCCGGCTTGACGCTGCATAAAAA | 334_A | TTTTTATGCAGCGTCAAGCCGGAATTTTTT |
| 335_S | AAAAAATTACTCCTAGCCCTCATTCAAAAA | 335_A | TTTTTGAATGAGGGCTAGGAGTAATTTTTT |
| 336_S | AAAAAATGGATCTTTACTTTAGGCCAAAAA | 336_A | TTTTTGGCCTAAAGTAAAGATCCATTTTTT |
| 337_S | AAAAAATCGCGTCCGTGCTCAATCCAAAAA | 337_A | TTTTTGGATTGAGCACGGACGCGATTTTTT |
| 338_S | AAAAAATGCTATCCGTGTCTATGCCAAAAA | 338_A | TTTTTGGCATAGACACGGATAGCATTTTTT |
| 339_S | AAAAAATGTGGTTACGGCCTCGATGAAAAA | 339_A | TTTTTCATCGAGGCCGTAACCACATTTTTT |
| 340_S | AAAAAATGTTTTTTAGAGGTGTGCAAAAAA | 340_A | TTTTTTGCACACCTCTAAAAAACATTTTTT |
| 341_S | AAAAAATTCGGGACTAGTAGTGGTGAAAAA | 341_A | TTTTTCACCACTACTAGTCCCGAATTTTTT |
| 342_S  linker | AAAAAAAAAAAAAAAAAAAAAAAAAAAAAA | 342_A  linker | TTTTTTTTTTTTTTTTTTTTTTTTTTTTTT |
| 343_S  linker | GGGGGGGGGGGGGGGGGGGGGGGGGGGGGG | 343_A  linker | CCCCCCCCCCCCCCCCCCCCCCCCCCCCCC |
| 344_S  W2(A/T) | AAAAAAATCAAAAGTTGACCAATAATAAAA | 344_A  W2(A/T) | TTTTATTATTGGTCAACTTTTGATTTTTTT |
| 345_S  W2m(A/T) | AAAAAAATCAAAAGTTacCCAATAATAAAA | 345_A  W2m(A/T) | TTTTATTATTGGgtAACTTTTGATTTTTTT |
| 346_S  W2(G/C) | GGGGGGATCAAAAGTTGACCAATAATGGGG | 346_A  W2(G/C) | CCCCATTATTGGTCAACTTTTGATCCCCCC |
| 347_S  W2m(G/C) | GGGGGGATCAAAAGTTacCCAATAATGGGG | 347_A  W2m(G/C) | CCCCATTATTGGgtAACTTTTGATCCCCCC |
