# Supplementary material for: Screening for Protein-DNA Interactions by Automatable DNA-Protein Interaction ELISA
Source: PLoS One. 2013 Oct 11;8(10):e75177. doi: 10.1371/journal.pone.0075177 (PMC3795721; doi:10.1371/journal.pone.0075177)
Supplement: Table S2 — Genes of interest. (DOCX) [file pone.0075177.s005.docx]

**Supporting Table S2|** Genes of interest. In grey gene specific primers for PCR.

| **AGI code** | **Gene name** | **Coding sequence** |
| --- | --- | --- |
| AT2G38470 | WRKY33 cDBD | atgagagagccgagaatcgtagtgcagacaacgagtgatattgacattcttgacgacggttacagatggagaaaatacggccagaaagtcgttaagggaaacccaaatccaagaagctactacaagtgcacaaccatcggttgtccagtgaggaaacatgtggagagagcatcacacgacatgagagcagtaatcacaacctacgaagggaaacacaaccacgatgttcctgcagctcgtggtagcggttacgccacaaacagagcaccacaggattcgtcttcagtcccgattagaccagctgctattgctggt |
| AT4G31550 | WRKY11 DBD | atgaagagaaccgtgagagtaccggcgataagtgcaaagatcgccgatattccaccggacgaatattcgtggaggaagtacggacaaaaaccgatcaagggctcaccacacccacgtggttactacaagtgcagtacattcagaggatgtccagcgaggaaacacgtggaacgagcattagatgatccagcgatgcttattgtgacatacgaaggagagcaccgtcataaccaatccgcgatgcaggagaatatttcttcttcaggcattaatgatttagtgtttgcctcggct |
| AT5G28770 | bZIP63  (supplied from Kichler et al. 2010) | atgagccatattcaacgggaaacgtcgaggccgcgattaaattccaacatggatgctgatttatatgggtataaatgggctcgcgataatgtcgggcaatcaggtgcgacaatctatcgcttgtatgggaagcccgatgcgccagagttgtttctgaaacatggcaaaggtagcgttgccaatgatgttacagatgagatggtcagactaaactggctgacggaatttatgcctcttccgaccatcaagcattttatccgtactcctgatgatgcatggttactcaccactgcgatccccggaaaaacagcattccaggtattagaagaatatcctgattcaggtgaaaatattgttgatgcgctggcagtgttcctgcgccggttgcattcgattcctgtttgtaattgtccttttaacagcgatcgcgtatttcgtctcgctcaggcgcaatcacgaatgaataacggtttggttgatgcgagtgattttgatgacgagcgtaatggctggcctgttgaacaagtctggaaagaaatgcataaacttttgccattctcaccggattcagtcgtcactcatggtgatttctcacttgataaccttatttttgacgaggggaaattaataggttgtattgatgttggacgagtcggaatcgcagaccgataccaggatcttgccatcctatggaactgcctcggtgagttttctccttcattacagaaacggctttttcaaaaatatggtattgataatcctgatatgaataaattgcagtttcatttgatgctcgatgagtttttctaa |
| AT4G24470 | TIFY1 | ATGTTTGGTCGCCATTCGATTATCCCAAATAACCAGATTGGTACCGCCTCTGCTTCCGCTGGTGAAGACCATGTCTCTGCCTCCGCTACGTCTGGTCACATTCCTTACGACGATATGGAAGAAATCCCTCATCCTGACTCTATCTATGGTGCTGCCTCCGATTTGATTCCCGATGGCTCTCAATTGGTTGCTCACCGATCCGATGGCTCTGAATTACTTGTTTCTCGGCCACCGGAAGGGGCGAATCAGCTTACGATCTCGTTCCGTGGACAAGTTTACGTTTTTGATGCCGTTGGTGCTGACAAGGTGGATGCTGTGTTGTCGCTGTTGGGTGGTTCTACTGAGCTTGCTCCTGGTCCGCAGGTGATGGAACTAGCTCAACAGCAGAATCATATGCCTGTTGTAGAATATCAGAGCCGCTGTAGCCTTCCGCAACGGGCACAATCCTTGGATAGGTTTCGGAAGAAGAGGAATGCTAGATGTTTCGAGAAGAAAGTAAGATACGGTGTTCGCCAAGAAGTTGCCTTAAGAATGGCACGTAATAAAGGTCAATTCACCTCTTCAAAGATGACAGATGGGGCTTATAACTCTGGCACAGATCAAGATTCTGCCCAAGATGATGCCCATCCAGAAATATCGTGTACTCATTGCGGCATTAGTTCCAAATGTACACCAATGATGCGACGTGGCCCTTCCGGCCCCAGGACTCTCTGCAATGCCTGTGGACTTTTTTGGGCTAACAGGGGTACATTGAGGGATCTCTCAAAGAAAACAGAAGAGAATCAGTTGGCTTTAATGAAACCGGATGATGGTGGGAGTGTTGCTGATGCTGCTAACAACTTAAACACTGAAGCTGCAAGTGTTGAAGAACACACTTCCATGGTTTCTCTTGCCAATGGGGATAATTCTAATCTGTTAGGTGATCAC |
| AT4G24470 | TIFY1 DBD | ATGGCCCATCCAGAAATATCGTGTACTCATTGCGGCATTAGTTCCAAATGTACACCAATGATGCGACGTGGCCCTTCCGGCCCCAGGACTCTCTGCAATGCCTGTGGACTTTTTTGGGCTAACAGGGGTACATTGAGGGATCTCTCAAAGAAAACAGAAGAGAATCAGTTGGCTTTAATGAAACCGGATGATGGTGGGAGTGTTGCTGATGCTGCTAACAACTTAAACACTGAAGCTGCAAGTGTTGAAGAACACACTTCCATGGTTTCTCTTGCCAATGGGGATAATTCTAATCTGTTAGGTGATCAC |
