# Supplementary material for: Screening for Protein-DNA Interactions by Automatable DNA-Protein Interaction ELISA
Source: PLoS One. 2013 Oct 11;8(10):e75177. doi: 10.1371/journal.pone.0075177 (PMC3795721; doi:10.1371/journal.pone.0075177)
Supplement: Table S3 — Raw absorbance data of WRKY11 DBD replicates. (DOCX) [file pone.0075177.s006.docx]

**Supporting Table S3|** Raw absorbance data of WRKY11 DBD replicates.

positively ranked dsDNA probes probed with WRKY11 DBD;

controls probes with WRKY11DBD;

controls probed with empty vector control.

| **Name** | **WRKY11 DBD #1** | **WRKY11 DBD #2** | **Name** | **WRKY11 DBD #1** | **WRKY11 DBD #2** | **Name** | **WRKY11 DBD #1** | **WRKY11 DBD #2** |
| --- | --- | --- | --- | --- | --- | --- | --- | --- |
| **1** | 0,54 | 0,65 | **46** | 0,73 | 0,54 | **91** | 3,74 | 3,19 |
| **2** | 0,57 | 0,52 | **47** | 0,85 | 0,62 | **92** | 2,17 | 1,65 |
| **3** | 0,6 | 0,51 | **48** | 0,76 | 0,55 | **93** | 0,74 | 0,54 |
| **4** | 0,56 | 0,49 | **49** | 0,57 | 0,42 | **94** | 1,04 | 0,64 |
| **5** | 0,65 | 0,55 | **50** | 0,59 | 0,45 | **95** | 0,79 | 0,58 |
| **6** | 0,64 | 0,56 | **51** | 0,63 | 0,46 | **96** | 1,03 | 0,58 |
| **7** | 0,74 | 0,65 | **52** | 0,6 | 0,46 | **97** | 3,61 | 3,4 |
| **8** | 0,71 | 0,62 | **53** | 0,69 | 0,51 | **98** | 0,61 | 0,47 |
| **9** | 0,45 | 0,31 | **54** | 1,56 | 1,18 | **99** | 0,62 | 0,55 |
| **10** | 0,58 | 0,42 | **55** | 0,81 | 0,59 | **100** | 0,63 | 0,53 |
| **11** | 0,7 | 0,51 | **56** | 0,76 | 0,6 | **101** | 1,22 | 2,08 |
| **12** | 0,59 | 0,44 | **57** | 0,65 | 0,5 | **102** | 0,64 | 0,58 |
| **13** | 0,65 | 0,51 | **58** | 0,64 | 0,46 | **103** | 0,78 | 0,68 |
| **14** | 0,67 | 0,58 | **59** | 0,64 | 0,47 | **104** | 0,7 | 0,6 |
| **15** | 0,8 | 0,65 | **60** | 0,65 | 0,47 | **105** | 0,5 | 0,34 |
| **16** | 0,75 | 0,6 | **61** | 0,71 | 0,54 | **106** | 0,66 | 0,46 |
| **17** | 0,58 | 0,43 | **62** | 0,78 | 0,58 | **107** | 0,81 | 0,52 |
| **18** | 0,58 | 0,44 | **63** | 0,81 | 0,57 | **108** | 1,74 | 1,11 |
| **19** | 0,6 | 0,44 | **64** | 0,75 | 0,58 | **109** | 0,81 | 0,63 |
| **20** | 0,61 | 0,45 | **65** | 0,58 | 0,42 | **110** | 0,8 | 0,65 |
| **21** | 0,71 | 0,58 | **66** | 0,62 | 0,55 | **111** | 0,86 | 0,66 |
| **22** | 0,71 | 0,63 | **67** | 0,64 | 0,57 | **112** | 0,79 | 0,59 |
| **23** | 0,76 | 0,62 | **68** | 0,58 | 0,55 | **113** | 0,64 | 0,46 |
| **24** | 0,81 | 0,57 | **69** | 0,73 | 0,62 | **114** | 0,68 | 0,47 |
| **25** | 0,8 | 0,75 | **70** | 0,81 | 0,57 | **115** | 0,63 | 0,48 |
| **26** | 0,55 | 0,43 | **71** | 0,81 | 0,65 | **116** | 0,68 | 0,49 |
| **27** | 0,68 | 0,45 | **72** | 0,76 | 0,61 | **117** | 0,81 | 0,61 |
| **28** | 0,59 | 0,45 | **73** | 0,55 | 0,35 | **118** | 0,79 | 0,6 |
| **29** | 0,64 | 0,54 | **74** | 0,65 | 0,48 | **119** | 0,84 | 0,64 |
| **30** | 0,7 | 0,61 | **75** | 0,73 | 0,53 | **120** | 0,87 | 0,57 |
| **31** | 0,73 | 0,63 | **76** | 0,68 | 0,46 | **121** | 0,62 | 0,45 |
| **32** | 0,74 | 0,57 | **77** | 0,71 | 0,53 | **122** | 0,66 | 0,48 |
| **33** | 0,42 | 0,34 | **78** | 0,76 | 0,54 | **123** | 0,68 | 0,49 |
| **34** | 0,56 | 0,47 | **79** | 0,81 | 0,64 | **124** | 0,67 | 0,5 |
| **35** | 0,69 | 0,64 | **80** | 0,97 | 0,6 | **125** | 0,75 | 0,56 |
| **36** | 0,85 | 0,95 | **81** | 0,58 | 0,43 | **126** | 0,8 | 0,59 |
| **37** | 0,6 | 0,52 | **82** | 0,61 | 0,46 | **127** | 0,74 | 0,59 |
| **38** | 0,62 | 0,53 | **83** | 0,69 | 0,39 | **128** | 0,85 | 0,55 |
| **39** | 0,71 | 0,66 | **84** | 0,65 | 0,48 | **129** | 0,43 | 0,36 |
| **40** | 0,73 | 0,59 | **85** | 0,72 | 0,53 | **130** | 0,59 | 0,5 |
| **41** | 0,5 | 0,34 | **86** | 0,9 | 0,68 | **131** | 0,65 | 0,54 |
| **42** | 0,62 | 0,49 | **87** | 0,76 | 0,57 | **132** | 0,58 | 0,53 |
| **43** | 0,81 | 0,66 | **88** | 0,94 | 0,64 | **133** | 0,66 | 0,56 |
| **44** | 0,68 | 0,5 | **89** | 0,64 | 0,44 | **134** | 0,69 | 0,59 |
| **45** | 0,73 | 0,57 | **90** | 0,75 | 0,46 | **135** | 0,81 | 0,71 |
| **136** | 0,68 | 0,61 | **184** | 0,8 | 0,59 | **232** | 0,76 | 0,61 |
| **137** | 3,56 | 2,66 | **185** | 0,61 | 0,44 | **233** | 2,15 | 1,54 |
| **138** | 0,75 | 0,46 | **186** | 0,67 | 0,48 | **234** | 0,65 | 0,48 |
| **139** | 0,72 | 0,53 | **187** | 0,75 | 0,5 | **235** | 0,73 | 0,56 |
| **140** | 0,65 | 0,48 | **188** | 0,83 | 0,5 | **236** | 0,72 | 0,49 |
| **141** | 0,9 | 0,54 | **189** | 0,97 | 0,58 | **237** | 0,76 | 0,56 |
| **142** | 0,78 | 0,56 | **190** | 0,87 | 0,61 | **238** | 0,75 | 0,58 |
| **143** | 0,94 | 0,64 | **191** | 0,86 | 0,6 | **239** | 1,54 | 1,46 |
| **144** | 0,88 | 0,59 | **192** | 0,94 | 0,56 | **240** | 0,99 | 0,59 |
| **145** | 0,58 | 0,47 | **193** | 0,44 | 0,37 | **241** | 1,55 | 0,45 |
| **146** | 0,63 | 0,47 | **194** | 0,64 | 0,52 | **242** | 0,64 | 0,49 |
| **147** | 0,65 | 0,49 | **195** | 3,26 | 2,66 | **243** | 0,69 | 0,51 |
| **148** | 0,66 | 0,5 | **196** | 0,82 | 0,73 | **244** | 0,65 | 0,49 |
| **149** | 0,86 | 0,55 | **197** | 1,21 | 0,97 | **245** | 0,71 | 0,56 |
| **150** | 0,84 | 0,62 | **198** | 1,48 | 1,51 | **246** | 1,75 | 1,15 |
| **151** | 1,28 | 0,59 | **199** | 0,81 | 0,69 | **247** | 0,76 | 0,59 |
| **152** | 0,79 | 0,55 | **200** | 0,89 | 0,62 | **248** | 0,77 | 0,56 |
| **153** | 0,58 | 0,48 | **201** | 0,46 | 0,37 | **249** | 0,58 | 0,44 |
| **154** | 0,63 | 0,49 | **202** | 3,61 | 2,56 | **250** | 0,64 | 0,48 |
| **155** | 0,69 | 0,54 | **203** | 0,71 | 0,56 | **251** | 0,73 | 0,53 |
| **156** | 0,62 | 0,5 | **204** | 0,72 | 0,51 | **252** | 0,7 | 0,5 |
| **157** | 0,72 | 0,54 | **205** | 0,79 | 0,58 | **253** | 0,8 | 0,56 |
| **158** | 0,79 | 0,61 | **206** | 0,81 | 0,58 | **254** | 0,83 | 0,63 |
| **159** | 0,75 | 0,58 | **207** | 0,93 | 0,7 | **255** | 0,78 | 0,61 |
| **160** | 0,87 | 0,63 | **208** | 0,72 | 0,62 | **256** | 0,82 | 0,57 |
| **161** | 0,74 | 0,75 | **209** | 0,59 | 0,47 | **257** | 0,45 | 0,39 |
| **162** | 0,56 | 0,49 | **210** | 0,61 | 0,49 | **258** | 0,61 | 0,48 |
| **163** | 0,64 | 0,55 | **211** | 0,65 | 0,5 | **259** | 0,6 | 0,58 |
| **164** | 0,62 | 0,66 | **212** | 0,7 | 0,51 | **260** | 3,44 | 3,47 |
| **165** | 0,66 | 0,56 | **213** | 0,74 | 0,58 | **261** | 1,16 | 1,14 |
| **166** | 0,68 | 0,59 | **214** | 0,79 | 0,64 | **262** | 0,69 | 0,59 |
| **167** | 0,81 | 0,68 | **215** | 0,83 | 0,61 | **263** | 0,78 | 0,68 |
| **168** | 0,71 | 0,63 | **216** | 0,78 | 0,59 | **264** | 0,74 | 0,64 |
| **169** | 0,59 | 0,39 | **217** | 0,57 | 0,45 | **265** | 0,57 | 0,43 |
| **170** | 0,68 | 0,46 | **218** | 0,66 | 0,49 | **266** | 0,69 | 0,52 |
| **171** | 0,69 | 0,54 | **219** | 0,66 | 0,52 | **267** | 0,73 | 0,56 |
| **172** | 0,66 | 0,49 | **220** | 0,64 | 0,52 | **268** | 0,69 | 0,54 |
| **173** | 3,61 | 3,19 | **221** | 0,68 | 0,57 | **269** | 0,76 | 0,56 |
| **174** | 0,76 | 0,56 | **222** | 1,2 | 1,08 | **270** | 0,75 | 0,57 |
| **175** | 0,83 | 0,68 | **309** | 0,65 | 0,56 | **338** | 0,59 | 0,49 |
| **176** | 0,99 | 0,62 | **310** | 0,77 | 0,64 | **339** | 0,64 | 0,49 |
| **177** | 0,65 | 0,45 | **223** | 0,78 | 0,61 | **340** | 0,63 | 0,51 |
| **178** | 0,66 | 0,48 | **224** | 0,71 | 0,61 | **341** | 0,66 | 0,55 |
| **179** | 1,39 | 0,5 | **225** | 0,43 | 0,37 | **271** | 1,52 | 1,25 |
| **180** | 0,74 | 0,49 | **226** | 0,59 | 0,51 | **272** | 0,91 | 0,62 |
| **181** | 0,72 | 0,56 | **227** | 0,65 | 0,57 | **273** | 0,62 | 0,44 |
| **182** | 1,17 | 0,91 | **228** | 0,64 | 0,5 | **274** | 0,59 | 0,47 |
| **183** | 0,78 | 0,63 | **229** | 0,65 | 0,59 | **275** | 0,66 | 0,5 |
| **280** | 0,84 | 0,58 | **230** | 0,68 | 0,6 | **276** | 3,82 | 3,15 |
| **281** | 0,6 | 0,45 | **231** | 0,75 | 0,68 | **277** | 0,71 | 0,58 |
| **282** | 0,6 | 0,47 | **311** | 0,72 | 0,6 | **278** | 0,79 | 0,63 |
| **283** | 0,68 | 0,48 | **312** | 0,72 | 0,59 | **279** | 0,8 | 0,61 |
| **284** | 0,7 | 0,49 | **313** | 0,59 | 0,43 | **342** | 0,59 | 0,47 |
| **285** | 0,75 | 0,56 | **314** | 0,64 | 0,48 | **342** | 0,54 | 0,5 |
| **286** | 0,81 | 0,62 | **315** | 0,6 | 0,49 | **343** | 1,2 | 0,48 |
| **287** | 1,82 | 1,51 | **316** | 0,69 | 0,51 | **343** | 1,1 | 0,5 |
| **288** | 0,78 | 0,56 | **317** | 0,66 | 0,69 | **344** | 3,82 | 3,67 |
| **289** | 0,42 | 0,37 | **318** | 0,74 | 0,64 | **344** | 3,56 | 3,56 |
| **290** | 0,58 | 0,5 | **319** | 0,73 | 0,63 | **344** | 3,51 | 3,52 |
| **291** | 0,7 | 0,65 | **320** | 0,76 | 0,6 | **344** | 0,78 | 0,86 |
| **292** | 0,64 | 0,57 | **321** | 0,41 | 0,36 | **344** | 0,63 | 0,35 |
| **293** | 0,66 | 0,59 | **322** | 0,55 | 0,48 | **345** | 0,78 | 0,6 |
| **294** | 0,73 | 0,6 | **323** | 0,61 | 0,54 | **345** | 0,66 | 0,64 |
| **295** | 0,74 | 0,69 | **324** | 0,59 | 0,45 | **345** | 0,41 | 0,44 |
| **296** | 0,71 | 0,61 | **325** | 0,68 | 0,59 | **345** | 0,73 | 0,5 |
| **297** | 0,46 | 0,35 | **326** | 0,73 | 0,58 | **345** | 0,51 | 0,31 |
| **298** | 0,59 | 0,48 | **327** | 0,79 | 0,67 | **346** | 3,67 | 3,19 |
| **299** | 0,66 | 0,54 | **328** | 0,75 | 0,64 | **346** | 3,67 | 2,92 |
| **300** | 0,6 | 0,51 | **329** | 0,55 | 0,38 | **346** | 3,56 | 3,37 |
| **301** | 0,68 | 0,56 | **330** | 0,58 | 0,47 | **346** | 3,51 | 2,55 |
| **302** | 0,66 | 0,56 | **331** | 0,65 | 0,53 | **346** | 3,47 | 2,66 |
| **303** | 0,79 | 0,67 | **332** | 0,59 | 0,57 | **347** | 0,78 | 0,56 |
| **304** | 0,75 | 0,62 | **333** | 0,67 | 0,56 | **347** | 0,75 | 0,57 |
| **305** | 0,55 | 0,44 | **334** | 0,73 | 0,58 | **347** | 0,58 | 0,46 |
| **306** | 0,6 | 0,48 | **335** | 2,05 | 0,67 | **347** | 1,08 | 0,58 |
| **307** | 0,62 | 0,5 | **336** | 1,22 | 0,62 | **347** | 0,54 | 0,41 |
| **308** | 0,64 | 0,51 | **337** | 0,52 | 0,44 |  |  |  |
