# Supplementary material for: Screening for Protein-DNA Interactions by Automatable DNA-Protein Interaction ELISA
Source: PLoS One. 2013 Oct 11;8(10):e75177. doi: 10.1371/journal.pone.0075177 (PMC3795721; doi:10.1371/journal.pone.0075177)
Supplement: Table S4 — Positively ranked dsDNA probes of DPI-ELISA screens. (DOCX) [file pone.0075177.s007.docx]

**Supporting Table S4|** Positively ranked dsDNA probes of DPI-ELISA screens. Grey: core consensus, respective cores/probes were identified by DREME that result in the respective WebLogos.

| **Protein of Interest** | **Name of DNA probe** | **Sequence of DNA probe 5’** 🡪 **3’** | **Rel. norm. absorbance** |
| --- | --- | --- | --- |
| WRKY33 DNA-binding domain  (significance threshold  >1.9) | 101_S | AAAAAAGTCTAAGGACTTATAGCTGTAAAA | 2.37 |
|  | 101_A | TTTTACAGCTATAAGTCCTTAGACTTTTTT |  |
|  | 92_S | AAAAAAAGTCTGCGGCTGCAGGGCGGAAAA | 2.36 |
|  | 92_A | TTTTCCGCCCTGCAGCCGCAGACTTTTTTT |  |
|  | 108_S | AAAAAAGGCGTCTGACTGTAACTCGTAAAA | 2.35 |
|  | 108_A | TTTTACGAGTTACAGTCAGACGCCTTTTTT |  |
|  | 54_S | AAAAAAGTCCGCCCTTAATTCATATTAAAA | 2.35 |
|  | 54_A | TTTTAATATGAATTAAGGGCGGACTTTTTT |  |
|  | 222_S | AAAAAAGGCGGTCGTAGTTGTGGGAGAAAA | 2.35 |
|  | 222_A | TTTTCTCCCACAACTACGACCGCCTTTTTT |  |
|  | 276_S | AAAAAACATCGCTGTCGTTGACGTTGAAAA | 2.34 |
|  | 276_A | TTTTCAACGTCAACGACAGCGATGTTTTTT |  |
|  | 195_S | AAAAAATGGTCGTCGTACTCGTGGTCAAAA | 2.34 |
|  | 195_A | TTTTGACCACGAGTACGACGACCATTTTTT |  |
|  | 233_S | AAAAAAGGTCCAGTCCACGTCATCAGAAAA | 2.34 |
|  | 233_A | TTTTCTGATGACGTGGACTGGACCTTTTTT |  |
|  | 83_S | AAAAAATATGTAGCTGCCGTTACGCTAAAA | 2.34 |
|  | 83_A | TTTTAGCGTAACGGCAGCTACATATTTTTT |  |
|  | 260_S | AAAAAAGTATAACGTGTTGTTGACCGAAAA | 2.33 |
|  | 260_A | TTTTCGGTCAACAACACGTTATACTTTTTT |  |
|  | 271_S | AAAAAACGTTGAACCTGGGCCTCAACAAAA | 2.33 |
|  | 271_A | TTTTGTTGAGGCCCAGGTTCAACGTTTTTT |  |
|  | 239_S | AAAAAAGTCGTTACACGTATGCGTGCAAAA | 2.32 |
|  | 239_A | TTTTGCACGCATACGTGTAACGACTTTTTT |  |
|  | 173_S | AAAAAATACATTTGACTTTGGGTTTCAAAA | 2.31 |
|  | 173_A | TTTTGAAACCCAAAGTCAAATGTATTTTTT |  |
|  | 137_S | AAAAAAACTCTAGGTCAATATTATTCAAAA | 2.30 |
|  | 137_A | TTTTGAATAATATTGACCTAGAGTTTTTTT |  |
|  | 202_S | AAAAAAGATTGACTCGTCTTACTCGAAAAA | 2.30 |
|  | 202_A | TTTTTCGAGTAAGACGAGTCAATCTTTTTT |  |
|  | 97_S | AAAAAAAGTTTGACCTTGGCGTTCGTAAAA | 2.28 |
|  | 97_A | TTTTACGAACGCCAAGGTCAAACTTTTTTT |  |
|  | 91_S | AAAAAAAGTGGCCGCTAGTCAACTCAAAAA | 2.25 |
|  | 91_A | TTTTTGAGTTGACTAGCGGCCACTTTTTTT |  |
|  | 43_S | AAAAAAAGGTCATTGCTGCTGGTGTTAAAA | 2.19 |
|  | 43_A | TTTTAACACCAGCAGCAATGACCTTTTTTT |  |
|  | 36_S | AAAAAATTTGGTCTTCGTCCTCGCTTAAAA | 2.17 |
|  | 36_A | TTTTAAGCGAGGACGAAGACCAAATTTTTT |  |
|  | 246_S | AAAAAACTGACCGTCAGCGCCCACGCAAAA | 2.09 |
|  | 246_A | TTTTGCGTGGGCGCTGACGGTCAGTTTTTT |  |
|  | 160_S | AAAAAACGGTCGCGATCGTTAATGATAAAA | 2.05 |
|  | 160_A | TTTTATCATTAACGATCGCGACCGTTTTTT |  |
|  | 88_S | AAAAAATCAACCATTATGGTATTACTAAAA | 2.02 |
|  | 88_A | TTTTAGTAATACCATAATGGTTGATTTTTT |  |
|  | 57_S | AAAAAAAATTAGTCTCTTCGTCTCCCAAAA | 2.01 |
|  | 57_A | TTTTGGGAGACGAAGAGACTAATTTTTTTT |  |
|  | 117_S | AAAAAAATCAGCCTGACTCTGGGCGTAAAA | 2.01 |
|  | 117_A | TTTTACGCCCAGAGTCAGGCTGATTTTTTT |  |
| TIFY1  (significance threshold >1.42) | 282_S | AAAAAACAGGCCGATTCCGATCCCGGAAAA | 1.83 |
|  | 282_A | TTTTCCGGGATCGGAATCGGCCTGTTTTTT |  |
|  | 201_S | AAAAAAGATCTAGGCCTTGGGACTCAAAAA | 1.81 |
|  | 201_A | TTTTTGAGTCCCAAGGCCTAGATCTTTTTT |  |
|  | 32_S | AAAAAACCTACGATGGTTCTGATCTTAAAA | 1.80 |
|  | 32_A | TTTTAAGATCAGAACCATCGTAGGTTTTTT |  |
|  | 38_S | AAAAAATTTATACTTGATCCTGGCTTAAAA | 1.78 |
|  | 38_A | TTTTAAGCCAGGATCAAGTATAAATTTTTT |  |
|  | 234_S | AAAAAAGGTCTGGACGATCACCGGGAAAAA | 1.70 |
|  | 234_A | TTTTTCCCGGTGATCGTCCAGACCTTTTTT |  |
|  | 238_S | AAAAAATCGGGGATCCGGGCGGTTGCAAAA | 1.69 |
|  | 238_A | TTTTGCAACCGCCCGGATCCCCGATTTTTT |  |
|  | 15_S | AAAAAAATCCGATCGATGCATATATAAAA | 1.63 |
|  | 15_A | TTTTATATATGCATCGATCGGATTTTTTT |  |
|  | 305_S | AAAAAACGCTTTCAGTTTTGATCTTAAAAA | 1.55 |
|  | 305_A | TTTTTAAGATCAAAACTGAAAGCGTTTTTT |  |
|  | 130_S | AAAAAACGCAACTCCGGATGTGGGGTAAAA | 1.54 |
|  | 130_A | TTTTACCCCACATCCGGAGTTGCGTTTTTT |  |
|  | 204_S | AAAAAAGGAAGTACTGACGATCTGGAAAAA | 1.52 |
|  | 204_A | TTTTTCCAGATCGTCAGTACTTCCTTTTTT |  |
|  | 290_S | AAAAAATGTCTAGACTCGGACATTCGAAAA | 1.52 |
|  | 290_A | TTTTCGAATGTCCGAGTCTAGACATTTTTT |  |
|  | 314_S | AAAAAATTGGCACACATGATCTCGGGAAAA | 1.50 |
|  | 314_A | TTTTCCCGAGATCATGTGTGCCAATTTTTT |  |
|  | 343_S | GGGGGGGGGGGGGGGGGGGGGGGGGGGGGG | 1.48 |
|  | 343_A | CCCCCCCCCCCCCCCCCCCCCCCCCCCCCC |  |
|  | 160_S | AAAAAACGGTCGCGATCGTTAATGATAAAA | 1.47 |
|  | 160_A | TTTTATCATTAACGATCGCGACCGTTTTTT |  |
|  | 300_S | AAAAAACTCGCGCTTGCATCTGTGCGAAAA | 1.45 |
|  | 300_A | TTTTCGCACAGATGCAAGCGCGAGTTTTTT |  |
|  | 227_S | AAAAAAGGCTCCGGCCCCCAATCGCAAAAA | 1.44 |
|  | 227_A | TTTTTGCGATTGGGGGCCGGAGCCTTTTTT |  |
|  | 334_S | AAAAAATTCCGGCTTGACGCTGCATAAAAA | 1.43 |
|  | 334_A | TTTTTATGCAGCGTCAAGCCGGAATTTTTT |  |
|  |  |  |  |

| **Protein of Interest** | **Name of DNA probe** | **Sequence of DNA probe 5’** 🡪 **3’** | **Rel. norm. absorbance** |
| --- | --- | --- | --- |
| bZIP63  (significance threshold  >1.86) | 109_S | AAAAAATACGTCTACGTGGTTGTCGTAAAA | 2.98 |
|  | 109_A | TTTTACGACAACCACGTAGACGTATTTTTT |  |
|  | 202_S | AAAAAAGATTGACTCGTCTTACTCGAAAAA | 2.68 |
|  | 202_A | TTTTTCGAGTAAGACGAGTCAATCTTTTTT |  |
|  | 313_S | AAAAAATCGCGAACGTACGTCCGCGGAAAA | 2.62 |
|  | 313_A | TTTTCCGCGGACGTACGTTCGCGATTTTTT |  |
|  | 239_S | AAAAAAGTCGTTACACGTATGCGTGCAAAA | 2.62 |
|  | 239_A | TTTTGCACGCATACGTGTAACGACTTTTTT |  |
|  | 233_S | AAAAAAGGTCCAGTCCACGTCATCAGAAAA | 2.57 |
|  | 233_A | TTTTCTGATGACGTGGACTGGACCTTTTTT |  |
|  | 108_S | AAAAAAGGCGTCTGACTGTAACTCGTAAAA | 2.54 |
|  | 108_A | TTTTACGAGTTACAGTCAGACGCCTTTTTT |  |
|  | 124_S | AAAAAAACTCACGGCGTCACTATGGTAAAA | 2.46 |
|  | 124_A | TTTTACCATAGTGACGCCGTGAGTTTTTTT |  |
|  | 31_S | AAAAAATCCACGACTCAGCAGCTCTTAAAA | 2.32 |
|  | 31_A | TTTTAAGAGCTGCTGAGTCGTGGATTTTTT |  |
|  | 44_S | AAAAAAGTACATGCGTAGATAATGTTAAAA | 2.11 |
|  | 44_A | TTTTAACATTATCTACGCATGTACTTTTTT |  |
|  | 223_S | AAAAAAGGCCACGGTAACCCCTTTTGAAAA | 2.06 |
|  | 223_A | TTTTCAAAAGGGGTTACCGTGGCCTTTTTT |  |
|  | 64_S | AAAAAATCGGGCCCCGTTAGCACTCTAAAA | 2.04 |
|  | 64_A | TTTTAGAGTGCTAACGGGGCCCGATTTTTT |  |
|  | 276_S | AAAAAACATCGCTGTCGTTGACGTTGAAAA | 2.02 |
|  | 276_A | TTTTCAACGTCAACGACAGCGATGTTTTTT |  |
|  | 287_S | AAAAAACACGCCATTGACACTGGTAAAAAA | 1.99 |
|  | 287_A | TTTTTTACCAGTGTCAATGGCGTGTTTTTT |  |
|  | 294_S | AAAAAACGTGCGCGTGCATGTCATCGAAAA | 1.97 |
|  | 294_A | TTTTCGATGACATGCACGCGCACGTTTTTT |  |
|  | 246_S | AAAAAACTGACCGTCAGCGCCCACGCAAAA | 1.96 |
|  | 246_A | TTTTGCGTGGGCGCTGACGGTCAGTTTTTT |  |
|  | 110_S | AAAAAAGCGTAAGCCTTGACAGTCGTAAAA | 1.94 |
|  | 110_A | TTTTACGACTGTCAAGGCTTACGCTTTTTT |  |
|  | 270_S | AAAAAACATGTCGCACCTGTACTAACAAAA | 1.93 |
|  | 270_A | TTTTGTTAGTACAGGTGCGACATGTTTTTT |  |
|  | 259_S | AAAAAAGTAGGTGCTGATGCTGGCCGAAAA | 1.92 |
|  | 259_A | TTTTCGGCCAGCATCAGCACCTACTTTTTT |  |
|  | 153_S | AAAAAATGTATTCGTGTCCACTCCATAAAA | 1.89 |
|  | 153_A | TTTTATGGAGTGGACACGAATACATTTTTT |  |
