# Supplementary material for: Screening for Protein-DNA Interactions by Automatable DNA-Protein Interaction ELISA
Source: PLoS One. 2013 Oct 11;8(10):e75177. doi: 10.1371/journal.pone.0075177 (PMC3795721; doi:10.1371/journal.pone.0075177)
Supplement: Text S1 — Promoter sequences of plant one-hybrid reporter plasmids. Promoter sequences of plant one-hybrid reporter plasmids. Promoter sequences are given in 5′→3′ orientation. Library probe 38 and the mutated 38m2 probe are highlighted in red, the CaMV 35Smini sequence is highlighted in green. Sites for HindIII, XhoI, SalI and NcoI restriction are not highlighted. (DOCX) [file pone.0075177.s009.docx]

**Supporting Text S1**| Promoter sequences of plant one-hybrid reporter plasmids. Promoter sequences are given in 5’ 🡪 3’ orientation. Library probe 38 and the mutated 38m2 probe are highlighted in red, the CaMV 35Smini sequence is highlighted in green. Sites for HindIII, XhoI, SalI and NcoI restriction are not highlighted.

p4x38-35Smini-LUC-NOS

AAGCTTCTCGAGTTTATACTTGATCCTGGCTTTTTATACTTGATCCTGGCTTTTTATACTTGATCCTGGCTTTTTATACTTGATCCTGGCTTGTCGACCGCAAGACCCTTCCTCTATATAAGGAAGTTCATTTCATTTGGAGAGGACACGTGGCCACCATGG

p4x38m2-35Smini-LUC-NOS

AAGCTTCTCGAGTTTATACTTGCTCCTGGCTTTTTATACTTGCTCCTGGCTTTTTATACTTGCTCCTGGCTTTTTATACTTGCTCCTGGCTTGTCGACCGCAAGACCCTTCCTCTATATAAGGAAGTTCATTTCATTTGGAGAGGACACGTGGCCACCATGG

p35Smini-LUC-NOS

AAGCTTCTCGACCGCAAGACCCTTCCTCTATATAAGGAAGTTCATTTCATTTGGAGAGGACACGTGGCCACCATGG
